# Empirical evidence for biometal dysregulation in Parkinson’s disease from a systematic review and Bradford Hill analysis

**DOI:** 10.1038/s41531-022-00345-4

**Published:** 2022-06-27

**Authors:** Amr H. Abdeen, Benjamin G. Trist, Kay L. Double

**Affiliations:** Brain and Mind Centre and School of Medical Sciences (Neuroscience), Faculty of Medicine and Health, The University of Sydney, Camperdown, Sydney, NSW 2050 Australia

**Keywords:** Parkinson's disease, Parkinson's disease

## Abstract

The Bradford Hill model evaluates the causal inference of one variable on another by assessing whether evidence of the suspected causal variable aligns with a set of nine criteria proposed by Bradford Hill, each representing fundamental tenets of a causal relationship. The aim of this study was to use the Bradford Hill model of causation to assess the level of empirical evidence supporting our hypotheses that alterations to iron and copper levels, and iron- and copper-associated proteins and genes, contribute to Parkinson’s disease etiology. We conducted a systematic review of all available articles published to September 2019 in four online databases. 8437 articles matching search criteria were screened for pre-defined inclusion and exclusion criteria. 181 studies met study criteria and were subsequently evaluated for study quality using established quality assessment tools. Studies meeting criteria for moderate to high quality of study design (*n* = 155) were analyzed according to the Bradford Hill model of causation. Evidence from studies considered of high quality (*n* = 73) supported a causal role for iron dysregulation in Parkinson’s disease. A causal role for copper dysregulation in Parkinson’s disease was also supported by high quality studies, although substantially fewer studies investigated copper in this disorder (*n* = 25) compared with iron. The available evidence supports an etiological role for iron and copper dysregulation in Parkinson’s disease, substantiating current clinical trials of therapeutic interventions targeting alterations in brain levels of these metals in Parkinson’s disease.

## Introduction

Parkinson’s disease is the fastest growing neurological condition worldwide^1^. Classical motor symptoms characterizing Parkinson’s disease result from the progressive degeneration of dopaminergic neurons in the substantia nigra (SN)^1^. Marked alterations in the levels of the essential biometals, iron and copper, are strongly associated with the death of nigral dopamine neurons in this disorder^2,3^, however, evidence for a causal role for these changes in dopamine neuron death is difficult to obtain from clinical studies, as technical limitations prevent quantitative studies of metal levels and SN cell death in vivo. Quantitative data describing brain metal levels and cell survival are available post-mortem but are temporally limited to a cross-section of clinical populations and are therefore purely correlative in nature.

In 1965, medical epidemiologist and statistician Austin Bradford Hill proposed a model for examining the causal inference that the alteration of a specific factor underlies an observed effect^4^. The Bradford Hill model evaluates whether all available evidence supports or challenges nine distinct criteria proposed by Bradford Hill (Table 1), each designed to interrogate crucial aspects of a causal association. Sir Bradford Hill developed these criteria during his investigation of a perceived causal association between tobacco smoking and lung cancer^5^. More recently, this model was found to challenge the etiological contribution of protein aggregates in neurodegenerative disorders, and hypothesized alternative roles for these species in neurodegeneration^6^.

**Table 1:** Criteria proposed by Bradford Hill and the definitions used in the current work to evaluate evidence for causality between changes in iron and copper and Parkinson’s disease.

Criterion	Definition
Strength	Changes in iron and copper levels, associated genes, and metalloproteins present in the Parkinson’s disease SN compared with appropriate controls using valid methods and accounting for confounding factors.
Consistency	Multiple studies from different geographic regions and populations using a variety of methods demonstrate consistent associations between SN changes in iron and copper biometal levels, associated genes, and metalloproteins with the development/progression of Parkinson’s disease.
Specificity	Changes in iron and copper biometal levels, associated genes, and metalloproteins are specific to degenerating brain regions in Parkinson’s disease and are not found in these same brain regions in other diseases.
Plausibility	Changes in iron and copper biometal levels, associated genes, and metalloproteins impair biochemical pathways implicated in Parkinson’s disease and/or trigger known degenerative pathways in the Parkinson’s disease SN.
Biological gradient	The magnitude of change in iron and copper biometal levels, associated genes, and metalloproteins in the Parkinson’s disease SN is associated with the severity and progression of Parkinson’s disease.
Analogy	Changes in iron and copper biometal levels, associated genes, and metalloproteins are associated with cell loss in other neurodegenerative diseases.
Temporality	Cause preceding effect: changes in iron and copper biometal levels, associated genes, and metalloproteins in the Parkinson’s disease SN occur prior to the emergence of parkinsonism.
Experiment	Clinical interventions normalize iron or copper biometal levels, or the function/expression of associated genes and metalloproteins in the Parkinson’s disease SN, thus decreasing incidence, severity, or rate of progression of the disease.
Coherence	Cause-effect should be consistent across widely accepted evidence, without contradictions or discrepancies.

In this study, we systematically reviewed published investigations of iron and copper in Parkinson’s disease and assessed the empirical evidence for an etiological role of iron and copper dysregulation in Parkinson’s disease using the Bradford Hill model. Identification of a causal association between biometal dyshomeostasis and Parkinson’s disease may reveal potential biomarkers and identify tractable pathways for slowing, or halting, disease progression.

## Results

### Quality assessment

**Fig. 1:**
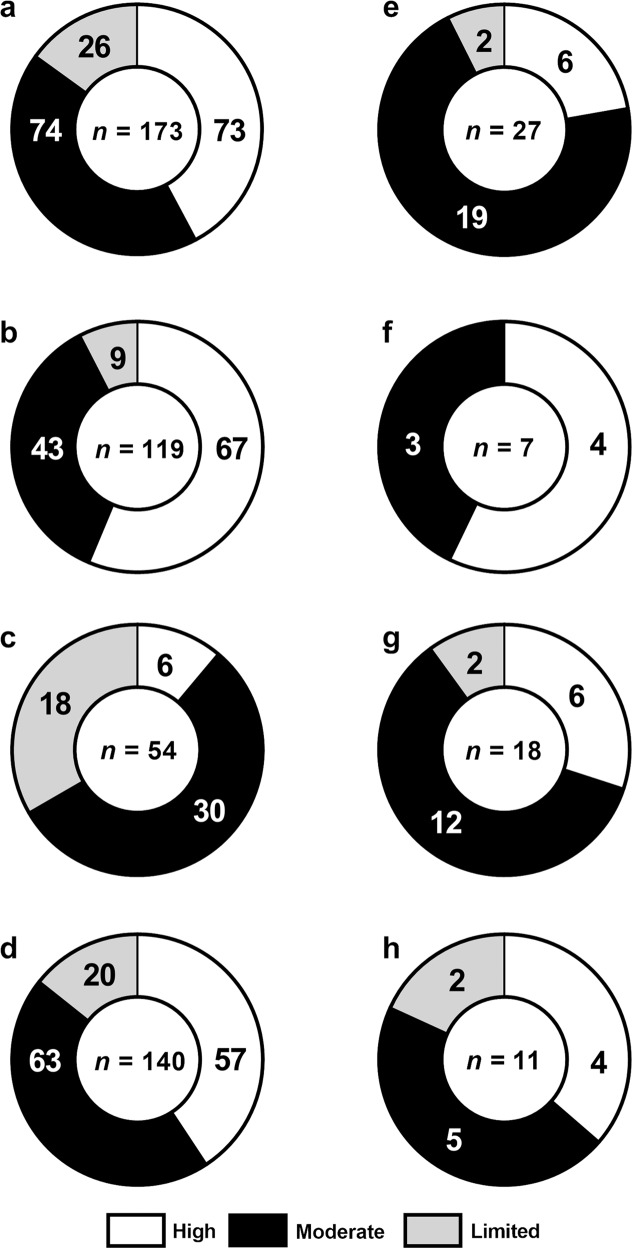
Distribution of the quality of included studies investigating the role of iron and copper pathways in Parkinson’s disease etiology. Study quality for articles investigating the role of iron (**a**–**d**) and copper (**e**–**h**) pathways in the pathogenesis of Parkinson’s disease quantified using the revised Genoud Scale, and NIH Quality Assessment Tools. Distribution of quality of all eligible articles (**a**, **e**), clinical studies (**b**, **f**), post-mortem investigations (**c**, **g**), and studies investigating the role of iron and copper metal only (**d**, **h**). Studies considered limited quality were excluded from further analyses.

Figure 1 illustrates the distribution of article quality according to the Genoud scale and NIH Quality Assessment Tools. Intrarater reliability using both scales were excellent (96% agreement, *n* = 27, *κ* = 0.926, *p* < 0.0001), whereas interrater reliability was substantial (89% agreement, *n* = 27, *κ* = 0.757, *p* < 0.0001).

### Assignment of studies investigating iron in Parkinson’s disease to Bradford Hill criteria

**Fig. 2:**
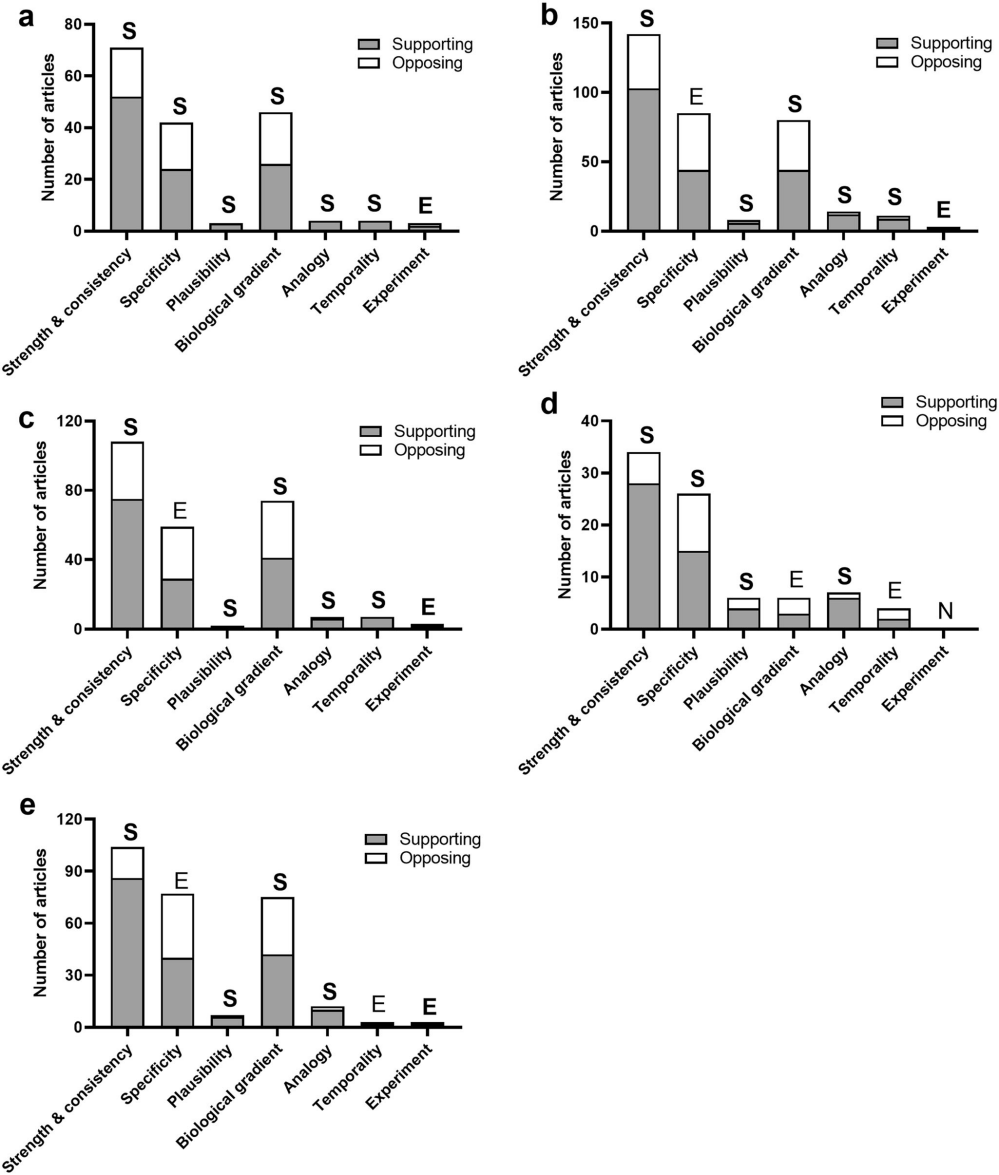
Bradford Hill criteria data and outcomes of all included studies investigating alterations in SN iron levels, or levels of ferroproteins or iron-associated genes in Parkinson’s disease. **a** Portrays results obtained from high quality articles alone, whereas **b** depicts the combined results from high and moderate quality articles. **c**, **d** Data obtained from clinical or post-mortem studies respectively. **e** Data obtained from all articles reporting alterations in iron metal levels in the Parkinson’s disease SN or SNc, that is, excluding articles reporting protein and/or genetic changes. Data for the Bradford Hill criterion, coherence, is considerably higher than other criteria and was therefore presented in Supplementary Table 2. Letters S (supporting), O (opposing), and E (equivocal) indicate overall criteria outcomes determined by analyses of all included studies. Non-bolded letters highlight discrepant criteria outcomes between each panel and (**a**). N, no data.

Following the assignment of high quality articles investigating iron in Parkinson’s disease (*n* = 76) to Bradford Hill criteria (Fig. 2a; Supplementary Tables 1 and 2), it was concluded that eight of the nine criteria (89%) support a causal association between iron dysregulation and Parkinson’s disease. The ninth criterion, *experiment*, was considered equivocal due to limited evidence (*n* = 3). Conclusions drawn from high quality studies were largely unchanged when additional data from moderate quality studies (*n* = 73) were included in the analysis (Fig. 2b), with the exception of the *specificity* criterion, which subsequently presented with an equal number of supporting and opposing studies.

Next, articles were divided into clinical and post-mortem investigations to assess whether conclusions drawn from these approaches differ. Evidence extracted solely from clinical studies (*n* = 110; Fig. 2c) were consistent with conclusions drawn from all studies (Fig. 2b), reflecting the preponderance of clinical studies investigating iron changes in Parkinson’s disease (*n* = 110 of total 146 studies, 75%). When post-mortem studies of iron were considered in isolation (*n* = 36; Fig. 2d), however, conclusions for four criteria conflicted with those drawn from clinical studies; *specificity* was supported, evidence for *biological gradient* and *temporality* were considered equivocal and there was no evidence to draw a valid conclusion for the *experiment* criterion.

To specifically address the role of altered iron metal levels in the etiology of Parkinson’s disease, we then excluded articles investigating iron-related proteins and genes (*n* = 36; Fig. 2e). Conclusions drawn from this subset of studies were identical to those derived from all studies, with the exception of *temporality*, which possessed insufficient evidence to draw a conclusion following the removal of protein and genetic studies. Overall, we conclude that the application of iron-related studies to the Bradford Hill criteria strongly supports a causal link between iron accumulation and Parkinson’s disease.

### Assignment of studies investigating copper in Parkinson’s disease to Bradford Hill criteria

Twenty-five full-text articles reporting quantitative studies of copper levels in the SN, and copper-associated pathways, in Parkinson’s disease (Supplementary Tables 1 and 3) were analyzed in an identical manner. The number of studies investigating alterations in nigral copper levels in Parkinson’s disease was substantially lower than those examining iron in this disorder, precluding definitive conclusions for some criteria. Almost two-thirds of the articles reporting copper data (*n* = 16 of 25 studies, 64%) also reported data for iron in the same samples.

**Fig. 3:**
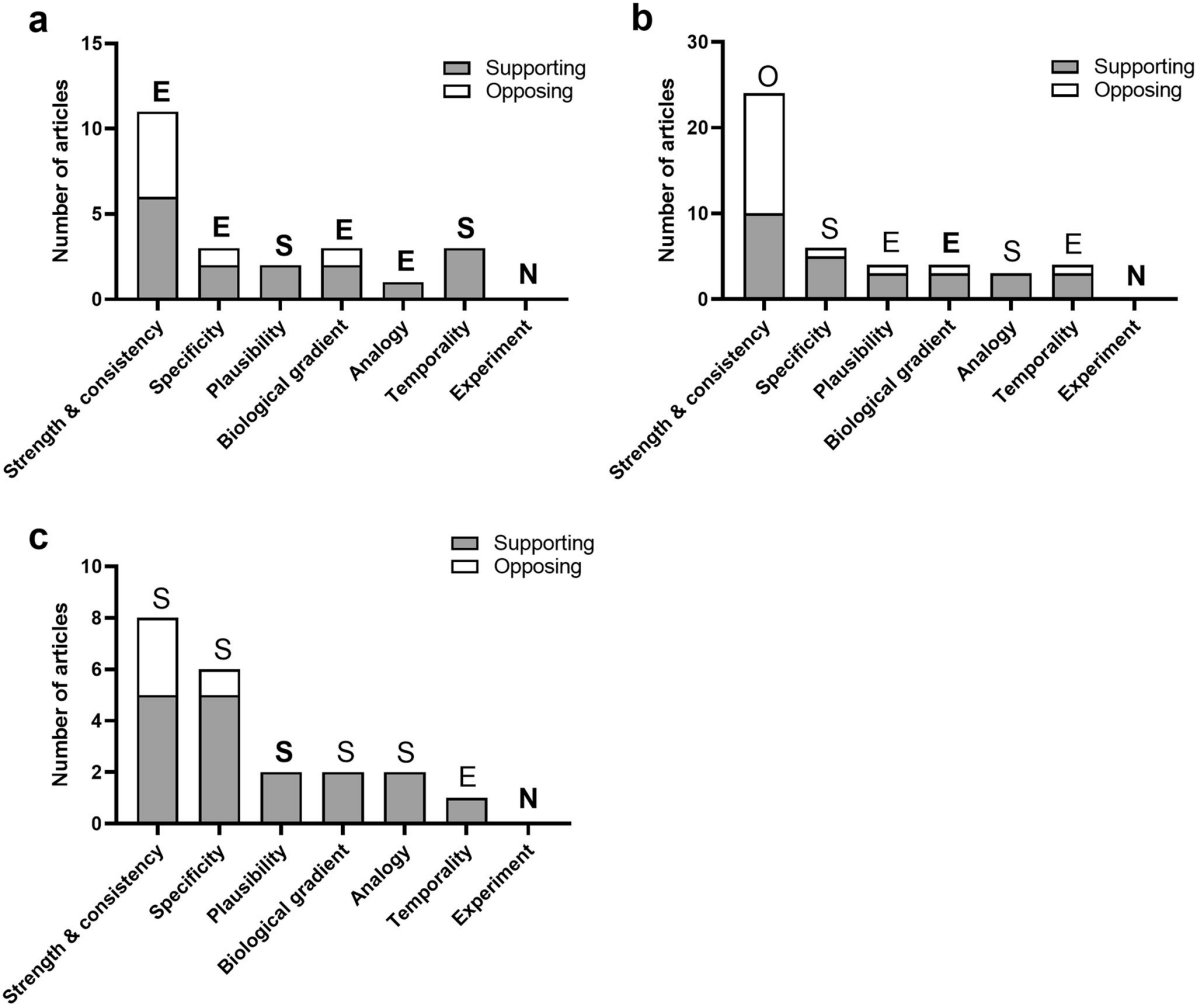
Bradford Hill criteria data and outcomes of all included articles investigating alterations in SN copper levels, or levels of cuproproteins or copper-associated genes in Parkinson’s disease. **a** Portrays results obtained from high quality articles alone, whereas **b** depicts the combined results from high and moderate quality articles. **c** All articles reporting alterations in copper metal levels in the Parkinson’s disease SN or SNc, that is, excluding articles reporting protein and/or genetic changes. Data for the Bradford Hill criterion, coherence, is considerably higher than other criteria and was therefore presented in Supplementary Table 3. Letters S (supporting), O (opposing), and E (equivocal) indicate overall criteria outcomes determined by analyses of all included studies. Non-bolded letters highlight discrepant criteria outcomes between each panel and (**a**). N, no data.

Evidence drawn from high quality studies investigating copper in Parkinson’s disease (*n* = 6) supported three of the nine Bradford Hill criteria (*plausibility, temporality, coherence*; Fig. 3a, Supplementary Table 3). Evidence for the remaining five criteria was considered equivocal, primarily because of the limited number of high quality studies addressing these criteria. No data was available for the ninth criterion, *experiment*, as there are yet no published articles on copper-modulating therapies in Parkinson’s disease patients. The addition of data from moderate quality studies (*n* = 19) subsequently allowed us to conclude that available data also support the criteria of *specificity, biological gradient and analogy* (Supplementary Table 3). In contrast, the addition of moderate quality studies to the remaining two criteria, *strength* and *consistency*, opposed a relationship between copper dysregulation and Parkinson’s disease.

Similar to iron, we sought to selectively interrogate the role of altered copper metal levels in Parkinson’s disease etiology by excluding articles investigating copper-related proteins and genes (*n* = 9; Fig. 3c). Seven of the nine Bradford Hill criteria were supported by this subgroup of articles, reinforcing the likelihood of an association between nigral copper deficiency and Parkinson’s disease. Limited data precluded definitive conclusions to be drawn for the remaining two criteria (*experiment* and *temporality*). Thus, available evidence most robustly supports a role for altered copper levels in Parkinson’s disease but not copper-associated proteins or genes. The number of studies investigating copper in Parkinson’s disease is substantially lower than those for iron, nevertheless, the Bradford Hill model supports a role for altered copper levels, but not copper-associated proteins or genes, in Parkinson’s disease.

### Systematic trends of quantitative metal analysis techniques in post-mortem Parkinson’s disease

Bulk metal analysis techniques provide a direct measurement of metal content in post-mortem tissue, in contrast to imaging-based techniques which provide an index of brain metal content. We therefore searched for systematic trends between findings from the two most common metal quantification techniques, inductively coupled plasma–mass spectrometry (ICP-MS) and absorption spectroscopy (AAS), used in post-mortem Parkinson’s disease tissue (Supplementary Table 4). Using ICP-MS, it was observed that *strength*, *consistency*, *specificity*, and *coherence* all demonstrated supporting conclusions for both iron and copper, although an insufficient number of studies was available to draw conclusions for the remaining criteria. In comparison, studies investigating the etiological role of iron in Parkinson’s disease demonstrated supporting conclusions for *strength, consistency, analogy*, and *coherence*, however, an equal number of supporting and opposing studies was seen for *specificity*. Remaining criteria presented with an insufficient number of studies to draw conclusions. Evidence for copper dyshomeostasis in Parkinson’s disease measured using AAS was available for three criteria only; *strength* and *consistency* were considered opposing, while *coherence* is opposing. Overall data obtained using ICP-MS, considered a superior quantitative method for metal analyses, supported the hypothesis that changes in iron and copper levels in the SN are causally associated with Parkinson’s disease at higher level compared to data drawn from studies using AAS.

Other techniques were also employed to quantify metal concentration in post-mortem Parkinson’s disease tissue, such as Perl’s stain, X-ray microanalysis, particle induced X-ray emission, and synchrotron X-ray fluorescence microscopy. According to our defined criteria, we were however unable to draw robust conclusions from these investigations due to the small number of studies using these methods (*n* ≤ 3).

### Relationship between iron dysregulation, disease duration, and UPDRS III motor scores

Parkinson’s disease is expressed over time as a well-characterized range of clinical disease stages, and therefore, we investigated if the empirical data collated in this study supported a relationship between disease duration, or severity, and iron dyshomeostasis. This analysis focused on clinical imaging studies of iron in Parkinson’s disease, given copper cannot currently be imaged in vivo and post-mortem tissues typically represent only late-stage disease.

To achieve this, we utilized the disease duration of patients involved in each study to classify them into early disease (ED, < 5 years; *n* = 95) or mid-late disease (MLD, ≥5 years; *n* = 80). Data from each group was then assessed independently as supporting or challenging each of the Bradford Hill criteria. Studies of early-stage disease patients supported six of the nine criteria, with the preponderance of evidence supporting *strength*, *consistency*, *specificity*, and *biological gradient*. The remaining three criteria either had insufficient (*analogy*, *temporality)* or equivocal evidence (*experiment)* of a role for iron dysregulation in Parkinson’s disease etiology. In contrast, MLD studies supported only three of the nine criteria—*strength, consistency*, and *coherence*. Interestingly, in MLD studies there was only equivocal evidence for *specificity* and *biological gradient*, suggesting that accumulation of iron in the Parkinson’s disease brain becomes more widespread in later disease stages. We were unable to draw any conclusions to the four remaining criteria as they were assigned either one, or no studies at all (Table 2).

**Table 2:** Bradford Hill analysis of studies investigating iron dysregulation in Parkinson’s disease after stratification according to disease duration.

Criteria	Outcome	Disease duration			
		ED	Conclusion	MLD	Conclusion
Strength and consistency	Supporting	31	Supporting	28	Supporting
	Opposing	4		4	
	Equivocal	0		2	
Specificity	Supporting	12	Supporting	9	Equivocal
	Opposing	7		9	
	Equivocal	5		2	
Plausibility	Supporting	2	Supporting	0	Insufficient data
	Opposing	0		0	
	Equivocal	0		0	
Biological gradient	Supporting	19	Supporting	11	Equivocal
	Opposing	7		11	
	Equivocal	3		2	
Analogy	Supporting	0	Insufficient data	1	Insufficient data
	Opposing	0		0	
	Equivocal	0		0	
Temporality	Supporting	1	Insufficient data	0	Insufficient data
	Opposing	0		0	
	Equivocal	0		0	
Experiment	Supporting	2	Equivocal	0	Insufficient data
	Opposing	1		0	
	Equivocal	0		0	
Coherence	Supporting	67	Supporting	49	Supporting
	Opposing	19		24	
	Equivocal	8		6	

Conclusions for individual criteria were drawn for early disease (ED; <5 years) and mid-late disease (MLD; ≥5 years) study cohorts.

In contrast to disease duration, the Unified Parkinson’s Disease Rating Scale (UPDRS) III motor scores provide an estimate of clinical disease severity, and therefore we repeated the above analysis using four patient cohorts stratified by UPDRS III motor scores (10.0–19.9; 20.0–29.9; 30.0–39.9; 40.0–49.9). Conclusions drawn from classifying studies according to disease severity were consistent with those from the analysis of disease duration; that is, studies support the criteria *strength* and *consistency* for iron accumulation in all clinical disease stages. Even though iron overload is specific to the Parkinson’s disease SN in cases with a UPDRS motor score under 30, more severe motor symptoms (UPDRS III 30.0–49.9) likely coincide with extranigral deposition of iron, evident from the equivocal conclusion drawn for *specificity*. Interestingly, the conclusions drawn for *biological gradient* are supporting in all subgroups, except for the category of scores ranging between 20.0 and 29.9, which was deemed equivocal, suggesting other regional pathologies may also contribute to clinical symptoms. Whilst *coherence* of the literature was supported across all subgroups, there was insufficient evidence to draw conclusions for the remaining criteria.

## Discussion

A large body of literature describes changes in iron and copper levels in the post-mortem Parkinson’s disease brain. In a meta-analysis of these works we demonstrated consistently and markedly increased iron levels, and an even greater change in the magnitude of copper deficiency, in the SN of Parkinson’s disease patients^3^. This data does not, however, provide information regarding a possible relationship between these changes and the dopamine neuron death characterizing this brain region in Parkinson’s disease. Thus, the question of a causal link between identified metal changes and the etiology of this disease remains unanswered. Here, we approach this question using the Bradford Hill model of causation. We demonstrate that data drawn from well-designed research studies overwhelmingly support the hypothesis that the increase in iron widely reported in the Parkinson SN is causally related to the disease process. Whilst limited data was available regarding copper changes in Parkinson’s disease, Bradford Hill stated that not all criteria needed to be addressed for his model to provide useful insights into causation. Hence, although we cannot draw any conclusions regarding copper with equivalent confidence, we find that available data also support a causal role for copper in the development of Parkinson’s disease.

The value of the current analysis lies in the identification of processes likely to be critical for disease progression and thus as rational targets for disease modification. An advantage of biometal levels as a pharmacological target is their tractable nature. Multiple iron-chelating compounds successfully mitigate neuronal loss in a range of murine and cellular models of brain iron dysregulation^7^, and initial data from human trials are promising. Deferiprone is a potent iron-chelating compound well tolerated at therapeutic doses (ClinicalTrials.gov identifier: NCT02728843), which effectively reduces iron content in the SN, other deep brain nuclei in Parkinson’s disease patients^8,9^. While existing technical limitations do not allow quantification of SN dopamine neuron number in deferiprone-treated patients, this study reported a significant reduction in motor disability determined by UPDRS III, which is reversed following cessation of deferiprone treatment^9^. These exciting data have prompted a phase two trial of deferiprone in *de novo* Parkinson’s disease (ClinicalTrials.gov identifier: NCT02655315), as well as a trial in amyotrophic lateral sclerosis (ALS; ClinicalTrials.gov identifier: NCT02655315) patients, another disorder where iron levels are proposed to be altered^10^. Data from these trials will fulfill Bradford Hill’s *experiment* criterion; however, the strongest evidence for a causal role for iron will require experimental studies of iron chelation in asymptomatic individuals progressing through the earliest stages of disease.

Early intervention with chelation therapy is likely to preserve the largest possible population of SN dopamine neurons, highlighting the importance of identifying Parkinson’s disease earlier than is possible at present. Prodromal biomarkers for Parkinson’s disease^11^ do not currently include brain metal changes; however, an abnormal sonographic marker referred to as nigral hyperechogenicity is an accepted risk marker, thought to reflect dysregulation of iron systems, including excess iron import and reduced intracellular export^12^. Nigral hyperechogenicity is reported in 90% of Parkinson’s disease patients up to 25 years prior to clinical diagnosis^13^, providing support to Bradford Hill’s criteria of *temporality*. In support of this, the available evidence supports the hypothesis that iron deposition is present in patients with mild disease (UPDRS III < 19.9) and ED SN pars compacta (SNc), suggesting disruptions to iron metabolism may be present prior to the onset of clinical symptoms. The association between altered iron levels in the SN and motor deficits in ED, but not MLD patients, (Table 2) suggests these changes may represent an etiologically important pathway for disease development. In contrast, the weak association reported between SN iron content and UPDRS III motor scores in patients with moderate-advanced motor deficits (UPDRS III score >30)^14,15^ suggests nigral iron accumulation is less closely linked to disease etiology in late stage disease when a range of secondary disease pathways are likely to be active.

Neuroimaging studies demonstrate significant correlations between nigral iron accumulation in Parkinson’s disease and parameters of disease severity and progression, including UPDRS and Hoehn–Yahr staging^16–19^. Interestingly, subgroup analysis of Parkinson’s disease patients revealed that these relationships are restricted to patients with akinetic/rigid-dominant disease^19,20^, suggesting bradykinesia and rigidity result from degeneration of the SN, whereas tremor is more closely associated with atrophy of extranigral structures. Magnetic resonance imaging techniques now provide us with reliable indices of iron content in human tissue and have since prompted investigations into understanding the spatial distribution of iron levels in the Parkinson’s disease brain and its association with symptomatic decline. Performing the Bradford Hill analysis on imaging studies, stratified according to disease duration (Table 2) and UPDRS III scores (Table 3), revealed that iron accumulation is specific to the SN during early stages of Parkinson’s disease, and is subsequently observed in extranigral structures, including the caudate nucleus^15,21–23^, globus pallidus^22,24–26^, putamen^15,21,22,24–28^, and red nucleus^23,25,26,29,30^, as the disease progresses.

**Table 3:** Bradford Hill analysis of studies investigating iron dysregulation in Parkinson’s disease after stratification according to Unified Parkinson’s Disease Rating Scale (UPDRS) III motor scores.

Criteria	Outcome	UPDRS III motor scores							
		10.0–19.9	Outcome	20.0–29.9	Outcome	30.0–39.9	Outcome	40.0–49.9	Outcome
Strength and consistency	Supporting	20	Supporting	18	Supporting	6	Supporting	2	Supporting
	Opposing	4		4		0		0	
	Equivocal	1		3		0		0	
Specificity	Supporting	9	Supporting	7	Supporting	0	Opposing	0	Opposing
	Opposing	6		4		2		2	
	Equivocal	1		3		1		1	
Plausibility	Supporting	0	Insufficient data	1	Insufficient data	0	Insufficient data	0	Insufficient data
	Opposing	0		0		0		0	
	Equivocal	0		0		0		0	
Biological gradient	Supporting	12	Supporting	8	Equivocal	4	Supporting	3	Supporting
	Opposing	6		8		2		0	
	Equivocal	3		2		0		0	
Analogy	Supporting	0	Insufficient data	0	Insufficient data	0	Insufficient data	0	Insufficient data
	Opposing	0		0		0		0	
	Equivocal	0		0		0		0	
Temporality	Supporting	0	Insufficient data	0	Insufficient data	0	Insufficient data	0	Insufficient data
	Opposing	0		0		0		0	
	Equivocal	0		0		0		0	
Experiment	Supporting	0	Insufficient data	2	Supporting	0	Insufficient data	0	Insufficient data
	Opposing	1		0		0		0	
	Equivocal	0		0		0		0	
Coherence	Supporting	41	Supporting	36	Supporting	10	Supporting	5	Supporting
	Opposing	17		16		4		2	
	Equivocal	5		8		1		1	

Conclusions for individual criteria were drawn for UPDRS III motor scores at a 10-point interval (10.0–19.9; 20.0–29.9; 30.0–39.9; 40.0–49.9).

We^31,32^, and others^33^, have argued that the unique interaction between labile iron and dopamine confers enhanced vulnerability to SN neurons in Parkinson’s disease. Heightened redox imbalance, fueled by the production of unstable dopamine metabolites, impairs cellular functions within these vulnerable neurons by forming adducts with proteins implicated in disease pathogenesis (e.g., α-synuclein, complex I, parkin)^31,32,34^. In addition, a cellular environment abundant in labile iron promotes the fibrillation and oligomerization of the ferrireductase α-synuclein, resulting in protein misfolding and inclusion formation whilst invoking the imbalance of ferric and ferrous iron^35^. The accumulation of iron is further accentuated by the perturbed function of key proteins involved in its metabolism^31,33^. Iron import into the cell is exaggerated due to the increased expression of lactoferrin receptors, which mediate the uptake of iron via lactoferrin. A secondary pathway triggered by nitrosative stress is driven by S-nitrosylation of DMT-1, and enhances the uptake of ferrous iron in the Parkinson’s disease SN^36^. Iron retention constitutes another pathway towards excess iron load. Neuronal iron export is reduced in the Parkinson’s disease SNc via a reduction in the ferroxidase activity of ceruloplasmin, possibly resulting from a copper deficit environment^37^, that normally mediates iron transport through ferroportin^37^. It is interesting to note that other investigations also revealed mutant variants of ceruloplasmin that are associated with a higher risk of Parkinson’s disease^38,39^, perhaps by exaggerating the extent of iron retention within neurons. Iron transport via ferroportin is also thought to be impaired in the Parkinson’s disease SNc, since the combined reduction in β-amyloid precursor protein and soluble tau destabilizes the structure of ferroportin at the cell surface^40–42^.

Our data cautiously support an etiological role for copper changes in Parkinson’s disease and restoration of brain copper levels is already under investigation as a therapeutic target. Originally developed as an imaging agent, copper(II) diacetylbis(N(4)-methylthiosemicarbazonato) (Cu^II^(atsm)) is suggested to target copper(II) delivery to brain regions experiencing oxidative stress and impaired mitochondrial function, changes reported in the Parkinson’s disease brain. Cu^II^(atsm) is reported to rescue SN neuronal loss, improve parkinsonian phenotype and prolong survival time in four distinct murine models of Parkinson’s disease^43^, although these models are not intended to recapitulate brain copper deficiency characteristic of the human disease. These investigations prompted the phase I clinical trials of Cu^II^(atsm) in idiopathic Parkinson’s disease (ClinicalTrials.gov Identifier: NCT03204929) and in ALS (ClinicalTrials.gov Identifier: NCT02870634). Initial evidence suggests daily treatment of early stage Parkinson’s disease patients with Cu^II^(atsm) for four months is well tolerated and is associated with clinically meaningful improvements in symptomology and quality of life^44^. Outcomes from these clinical trials will provide valuable evidence to assess the potential of copper supplementation in modulating clinical disease trajectory.

Interestingly, copper levels in the SN are two-fold higher than those in other regions of the healthy brain^45^, suggesting this region has an increased demand for, and a higher capacity to accumulate, this biometal. Using synchrotron X-ray fluorescence microscopy, we showed the copper content of individual catecholaminergic SN and locus coeruleus neurons are significantly reduced in a putative preclinical form of Parkinson’s disease, incidental Lewy body disease, suggesting this change is an early event in disease pathogenesis^45^. These data suggest that the regional decrease in copper early in the Parkinson’s disease brain may represent a biomarker for the disease process. In contrast to the accumulation of iron in multiple deep brain nuclei with varying levels of cell loss in the Parkinson’s disease brain, the marked copper deficiency is restricted to regions of neuronal degeneration. Elemental analysis of metal concentrations in a progressive form of parkinsonism known as Parkinson’s disease dementia demonstrated copper deficiency in seven out of nine investigated brain regions, whereas iron accumulation was not observed in any brain regions, highlighting the marked differences in patterns of defective metal levels during advanced stages of the disease^46^. Currently, a tool for in vivo quantification of brain copper is not available but such a technology would be valuable to elucidate the spatiotemporal nature of copper changes and its relationship to clinical disease.

The potential consequences of low brain copper levels can be inferred from analogous copper changes in other neurological disorders. In Menkes disease, a genetic loss-of-function mutation in the copper transport protein ATP7A results in a systemic copper deficiency, including in the central nervous system^47^. Motor dysfunction and neurodegeneration characteristic of Menkes disease are considered to result from inadequate copper loading, and subsequent dysfunction of, cuproproteins mediating essential biological functions for neuronal viability and survival^48^. We posit that impaired cuproprotein structure and function, resultant from copper deficiency, constitutes a fundamental pathway to the degeneration of vulnerable neuronal populations in phenotypically distinct disorders^49^.

Although in vitro studies have suggested that copper may contribute to disease etiology by stimulating oligomerization and deposition of α-synuclein, the depletion of cellular copper reserves in the Parkinsonian SN suggests that copper-mediated stimulation of synucleinopathy is unlikely to underlie neurodegeneration in this brain region. By contrast, we recently reported a wildtype Cu/Zn-superoxide dismutase (SOD1) proteinopathy associated with neuronal loss in the Parkinson’s disease SN closely resembling misfolded SOD1 linked to neuronal death in SOD1-associated ALS spinal cord^50,51^. The specificity of SOD1 misfolding and aggregation to copper-deficient brain regions is suggestive of perturbed copper delivery to other cuproproteins, especially those with a lower affinity to copper^52^. Despite this evidence, an opposing conclusion is drawn for Bradford Hill’s *strength* and *consistency* when considering SOD1 protein/gene on its own (Supplementary Table 5). This finding is influenced by the fact that four of these nine reported studies investigated specific mutations in *SOD1* in Parkinson’s disease patients which were not found to be present. While this suggests that a mutation in *SOD1* is unlikely to be causally linked to Parkinson’s disease it does not exclude other possible disease-linked mechanisms associated with this protein. The influence of genetic studies on Bradford Hill’s criteria is also seen in the case of ferritin, transferrin, hemeoxygenase-1 and ceruloplasmin (Supplementary Table 5). Indeed, this highlights the importance of Bradford Hill’s comment in his original work that none of the nine criteria provide indisputable evidence for or against causality; rather he stated that all available data must be considered in its totality^4^. In part, this is a contributing factor to our inability to identify the metalloprotein and/or gene that is most causally associated with Parkinson’s disease. More importantly, however, is that there is a limited number of studies that fulfilled individual criteria for the investigated proteins and genes, allowing us to draw conclusions for a maximum of five criteria for only a few of these proteins and genes (Supplementary Table 5). Further research into specific changes in individual metalloproteins and genes in Parkinson’s disease is therefore required to support an argument of causality for any one protein or gene using the Bradford Hill model.

Application of the Bradford Hill model of causation to published evidence of iron dysregulation strongly supports the hypothesis that this pathology plays a causal role in nigral neurodegeneration in Parkinson’s disease. More limited data is available for copper, but the available evidence cautiously supports an etiological role for this biometal in this disorder. Iron and copper are involved in a range of accepted degenerative pathways in Parkinson’s disease; it is feasible therefore that therapeutic modulation of either, or both, biometals may elicit widespread mechanisms of action with potential benefits for slowing disease progression.

## Methods

### Search strategy and study selection

**Fig. 4:**
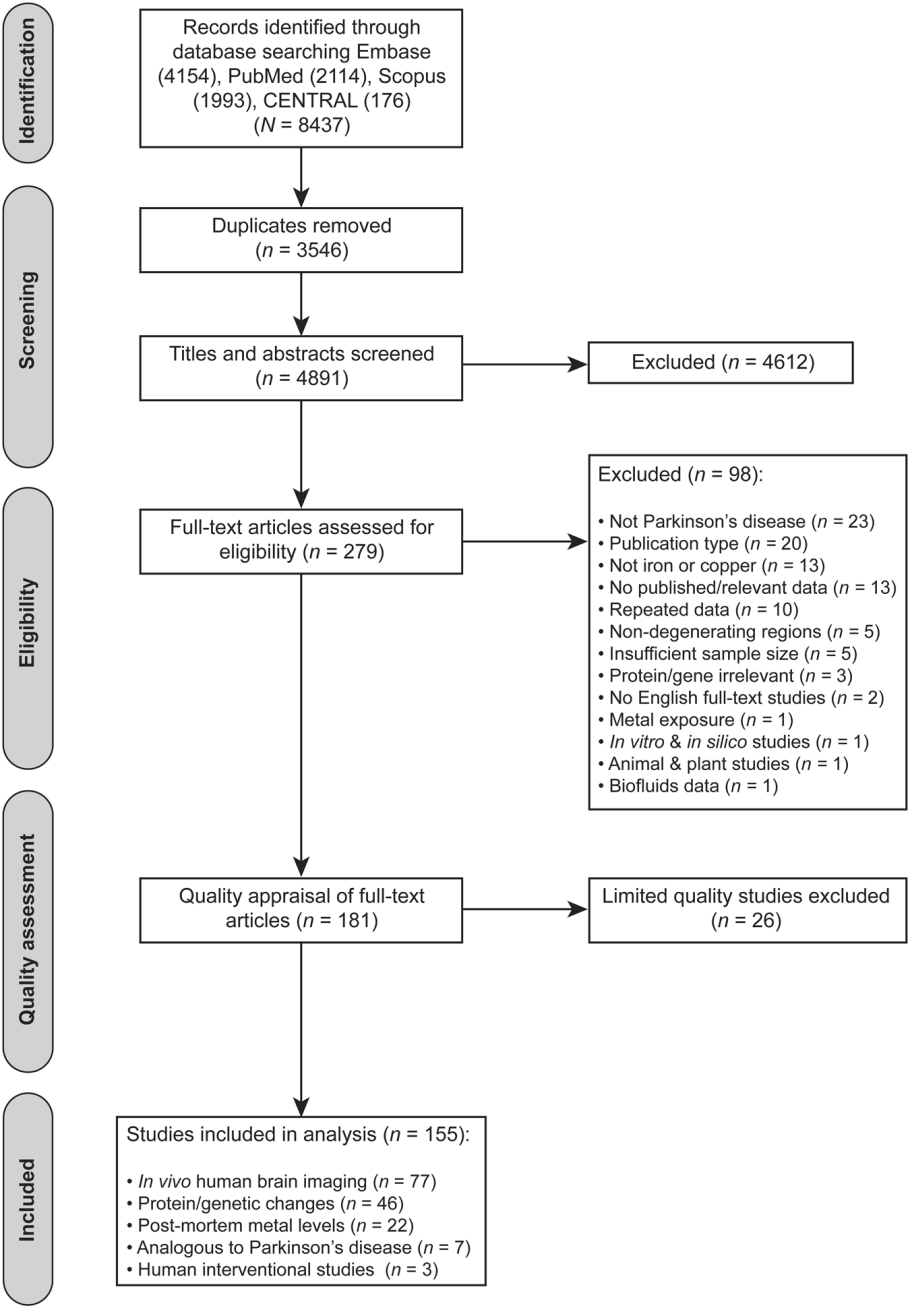
PRISMA flowchart of literature search, screening, eligibility, and quality assessment. CENTRAL The Cochrane Controlled Register of Controlled Trials.

To identify eligible studies for assignment to Bradford Hill criteria, hereafter termed Bradford Hill analysis, we conducted a systematic review according to the guidelines of the Preferred Reporting Items for Systematic Reviews and Meta Analyses (PRISMA) statement (Fig. 4). We performed an electronic search for research articles published online in PubMed, EMBASE, Cochrane Central Register of Controlled Trials (CENTRAL) and Scopus databases from inception until September 9th, 2019 using the terms “Parkinson*”, “*copper*”,” Cu”, “cupro*”, “*copper protein?*”, “*iron*”, “*Fe*”, “ferro*” and “*iron protein?*” (Supplementary Table 6). As Parkinson’s disease does not develop naturally in other species, a filter for human studies was applied to the database searches. In the case of Scopus, the term *“human”* was added to its search strategy as a filter function was not available at the date of the search. Our search strategy generated 8,437 records collectively from all four databases. Duplicates were removed using a 12-step process modified from published literature (Supplementary Table 7)^53^. Titles and abstracts of the remaining 4,891 unique articles were independently screened by two authors (A.A., B.T.) according to pre-defined inclusion and exclusion criteria (Supplementary Table 8). Eligible studies reported quantitative and semi-quantitative data of iron and/or copper levels in the Parkinson’s disease SN, investigated biochemical changes in the levels or function of predetermined genes and metalloproteins (Supplementary Table 9) in the Parkinson’s disease SN, evaluated the therapeutic potential of interventions altering brain metal levels in Parkinson’s disease patients, or investigated any of these variables of interest in regions of neuron death in other neurodegenerative disorders. Screening excluded a further 4612 studies. Complete texts of the remaining 279 studies were reviewed for eligibility, resulting in the exclusion of a further 98 studies that did not meet inclusion criteria (Fig. 4).

### Quality assessment and data extraction

The remaining 181 articles, comprising both clinical and post-mortem studies of Parkinson’s disease patients, were critically appraised using two independent scales to quantify study quality and bias. We recently developed a scale for the assessment of the quality of research studies of biochemical endpoints in post-mortem human tissue samples, the Quality Assessment Scale for Human Tissue, or briefly the Genoud Scale^3^. This scale scores aspects of study design, sample selection, tissue quality, detection method, and statistical analysis to provide a single metric of overall study quality. In the current work, we amended our previously published scale to account for the differential exclusion of outliers between experimental groups (details in Supplementary Table 10). We categorized study quality using predetermined discretionally values; studies scoring <50% were considered limited quality (*n* = 26 of total 181 studies, 14%) and were excluded from further analyses, whilst studies scoring 50–75% and >75% were deemed moderate and high quality, respectively. High and moderate quality studies were subsequently used for the Bradford Hill analysis. As the Genoud Scale has limited applicability for clinical studies, we assessed the quality of studies examining Parkinson’s disease patients using a modified version of the Study Quality Assessment Tools developed by the National Heart, Lung, and Blood Institute (National Heart, Lung, and Blood Institute Study Quality Assessment Tools). These Tools are designed to assess quality and bias in a broad variety of clinical study designs, including controlled intervention studies, observational and cross-sectional studies, as well as case-control studies. As these Tools were originally designed to be qualitative scales, we designated quantitative scores to individual criteria outcomes (yes = 1; no/cannot determine = 0; not applicable = excluded) to similarly provided a single quantitative metric for the quality of individual studies to enable comparison of study quality outcomes with those obtained using the Genoud Scale. For both scales, points awarded were expressed as a proportion of the maximum possible score.

The interrater and intrarater reliability of article quality assessment outcomes were calculated and statistically analyzed using Cohen’s kappa. Intrarater reliability was assessed by one author (A.A.), who applied both quality assessment scales to the same 15% of all eligible articles at two separate times. Interrater reliability was evaluated by two authors (A.A., B.T.), who independently applied both quality assessment scales to the same 20% of eligible articles at the same time point.

One hundred and fifty-five articles met all inclusion criteria and were subsequently individually examined for empirical data on iron or copper dysregulation—changes in the regional localization or concentration of iron or copper, as well as alterations to the levels or function of copper—or relevant proteins or genes, within the Parkinson’s disease brain (Supplementary Table 6). Data for the SNc was preferentially extracted as dopamine neurons are located in this subregion^31^; where this was not possible, data for the SN as a whole was used. Studies supported a given criterion if data was consistent with the definition in Table 1, or opposed a criterion if data challenged its definition. For example, a study reporting a statistically significant increase in nigral iron levels in Parkinson’s disease patients compared to controls, that were absent in other investigated brain regions, would be considered to support Bradford Hill’s *strength*, *consistency* and *specificity* criteria according to the definitions presented in Table 1. In contrast, a study presenting with no statistically significant changes in nigral iron levels in Parkinson’s disease compared with controls, would be considered to oppose Bradford Hill’s *strength* and *consistency*. An equivocal outcome would be drawn if the study presents conflicting evidence for one criterion; this can be illustrated by a study reporting multiple indices of iron levels in the brain, in which some indicate elevated iron levels whereas others demonstrate no changes. This process was completed for all included studies by two researchers (A.A., B.T.) in an independent manner. The number of studies providing supporting and opposing evidence for each criterion was subsequently calculated, with equivocal outcomes included as both a supporting and an opposing study for relevant criteria. This previously described method^6^ enabled us to empirically evaluate the available evidence from each study individually, while also accounting for the breadth of literature investigating the role of iron and copper dyshomeostasis in Parkinson’s disease (*n* = 155; Supplementary Table 1). Quantification of studies assigned to Bradford Hill criteria were subsequently used to establish the level of evidence for a causal association between metal dyshomeostasis and Parkinson’s disease.

A determination was made regarding whether the available data support or oppose each of the Bradford Hill criterion. For Bradford Hill criteria with data drawn from >15 total articles, a conclusion was only drawn if the summed number of articles for one outcome exceeded that of the other by greater than 20%. For criteria with ≤15 total articles, a conclusion was only drawn if the number of articles for one outcome was at least 2 studies greater than the alternative conclusion. If these conditions were not met, we concluded that the available data provides only equivocal evidence for both conclusions. Criteria with data available from <4 articles were considered to have limited evidence to draw robust conclusions. All figures were generated using GraphPad Prism 8.0.2.

## Supplementary information

Supplementary Material

## Data Availability

The datasets generated during and/or analyzed during the current study are available from the corresponding author on reasonable request. Review protocol can be accessed through the PROSPERO database (ID: CRD42020158895).

## References

[1] Bloem BR, Okun MS, Klein C (2021). Parkinson’s disease. Lancet.

[2] Barnham KJ, Bush AI (2014). Biological metals and metal-targeting compounds in major neurodegenerative diseases. Chem. Soc. Rev..

[3] Genoud S, Senior AM, Hare DJ, Double KL (2020). Meta-analysis of copper and iron in Parkinson’s disease brain and biofluids. Mov. Disord..

[4] Hill AB (2015). The environment and disease: Association or causation?. J. R. Soc. Med..

[5] Doll R, Hill AB (1950). Smoking and carcinoma of the lung: Preliminary report. Br. Med. J..

[6] Espay AJ (2019). Revisiting protein aggregation as pathogenic in sporadic Parkinson and Alzheimer diseases. Neurology.

[7] Devos D (2020). Conservative iron chelation for neurodegenerative diseases such as Parkinson’s disease and amyotrophic lateral sclerosis. J. Neural Transm..

[8] Martin-Bastida A (2017). Motor associations of iron accumulation in deep grey matter nuclei in Parkinson’s disease: a cross-sectional study of iron-related magnetic resonance imaging susceptibility. Eur. J. Neurol..

[9] Devos D (2014). Targeting chelatable iron as a therapeutic modality in Parkinson’s disease. Antioxid. Redox Signal..

[10] Ignjatović A, Stević Z, Lavrnić S, Daković M, Bačić G (2013). Brain iron MRI: A biomarker for amyotrophic lateral sclerosis. J. Magn. Reson. Imaging.

[11] Heinzel S (2019). Update of the MDS research criteria for prodromal Parkinson’s disease. Mov. Disord..

[12] Yu S (2018). Clinical features and dysfunctions of iron metabolism in Parkinson disease patients with hyper echogenicity in substantia nigra: A cross-sectional study. BMC Neurol..

[13] Postuma RB, Berg D (2016). Advances in markers of prodromal Parkinson disease. Nat. Rev. Neurol..

[14] Guan X (2015). Regionally progressive accumulation of iron in Parkinson’s disease as measured by quantitative susceptibility mapping. NMR Biomed..

[15] Hopes L (2016). Magnetic resonance imaging features of the nigrostriatal system: Biomarkers of Parkinson’s disease stages?. PLoS One.

[16] Guan X (2017). Influence of regional iron on the motor impairments of Parkinson’s disease: A quantitative susceptibility mapping study. J. Magn. Reson. Imaging.

[17] Homayoon N (2019). Nigral iron deposition in common tremor disorders. Mov. Disord..

[18] Du G (2016). Quantitative susceptibility mapping of the midbrain in Parkinson’s disease. Mov. Disord..

[19] An H (2018). Quantifying iron deposition within the substantia nigra of Parkinson’s disease by quantitative susceptibility mapping. J. Neurol. Sci..

[20] Jin L (2012). Nigral iron deposition occurs across motor phenotypes of Parkinson’s disease. Eur. J. Neurol..

[21] Antonini A (1993). T2 relaxation time in patients with Parkinson’s disease. Neurology.

[22] Uchida Y (2019). Voxel-based quantitative susceptibility mapping in Parkinson’s disease with mild cognitive impairment. Mov. Disord..

[23] Zhang W (2009). Determination of brain iron content in patients with Parkinson’s disease using magnetic susceptibility imaging. Neurosci. Bull..

[24] Bartzokis G (1999). MRI evaluation of brain iron in earlier- and later-onset Parkinson’s disease and normal subjects. Magn. Reson. Imaging.

[25] Qiao PF, Shi F, Jiang MF, Gao Y, Niu GM (2017). Application of high-field magnetic resonance imaging in Parkinson’s disease. Exp. Ther. Med..

[26] Wu SF (2014). Assessment of cerebral iron content in patients with Parkinson’s disease by the susceptibility-weighted MRI. Eur. Rev. Med. Pharmacol. Sci..

[27] Ulla M (2013). Is R2* a new MRI biomarker for the progression of Parkinson’s disease? A longitudinal follow-up. PLoS One.

[28] Wallis LI (2008). MRI assessment of basal ganglia iron deposition in Parkinson’s disease. J. Magn. Reson. Imaging.

[29] Chen Q (2019). Iron deposition in Parkinson’s disease by quantitative susceptibility mapping. BMC Neurosci..

[30] Shahmaei V, Faeghi F, Mohammdbeigi A, Hashemi H, Ashrafi F (2019). Evaluation of iron deposition in brain basal ganglia of patients with Parkinson’s disease using quantitative susceptibility mapping. Eur. J. Radiol. Open.

[31] Trist BG, Hare DJ, Double KL (2019). Oxidative stress in the aging substantia nigra and the etiology of Parkinson’s disease. Aging Cell.

[32] Hare DJ, Double KL (2016). Iron and dopamine: A toxic couple. Brain.

[33] Zucca FA (2017). Interactions of iron, dopamine and neuromelanin pathways in brain aging and Parkinson’s disease. Prog. Neurobiol..

[34] Schapira AHV (1990). Mitochondrial complex I deficiency in Parkinson’s disease. J. Neurochem..

[35] Sian-Hulsmann J, Riederer P (2020). The role of alpha-synuclein as ferrireductase in neurodegeneration associated with Parkinson’s disease. J. Neural Transm..

[36] Liu C (2018). S-nitrosylation of divalent metal transporter 1 enhances iron uptake to mediate loss of dopaminergic neurons and motoric deficit. J. Neurosci..

[37] Ayton S (2013). Ceruloplasmin dysfunction and therapeutic potential for Parkinson disease. Ann. Neurol..

[38] Hochstrasser H (2005). Functional relevance of ceruloplasmin mutations in Parkinson’s disease. FASEB J..

[39] Zhao N (2015). Single-nucleotide polymorphisms and haplotypes of non-coding area in the CP gene are correlated with Parkinson’s disease. Neurosci. Bull..

[40] Wong BX (2014). Β-Amyloid precursor protein does not possess ferroxidase activity but does stabilize the cell surface ferrous iron exporter ferroportin. PLoS One.

[41] Ayton S (2015). Parkinson’s disease iron deposition caused by nitric oxide-induced loss of beta-amyloid precursor protein. J. Neurosci..

[42] Lei P (2012). Tau deficiency induces parkinsonism with dementia by impairing APP-mediated iron export. Nat. Med..

[43] Hung LW (2012). The hypoxia imaging agent CuII(atsm) is neuroprotective and improves motor and cognitive functions in multiple animal models of Parkinson’s disease. J. Exp. Med..

[44] Evans, A., Rowe, D., Lee, W., Noel, K. & Rosenfeld, C. Preliminary evidence of CuATSM treatment benefit in Parkinson’s disease. *International Association of Parkinsonism and Related Disorders.* 16–19 Montreal, Canada (2019).

[45] Davies KM (2014). Copper pathology in vulnerable brain regions in Parkinson’s disease. Neurobiol. Aging.

[46] Scholefield M (2021). Widespread decreases in cerebral copper are common to Parkinson’s disease dementia and Alzheimer’s disease dementia. Front. Aging Neurosci..

[47] Madsen E, Gitlin JD (2007). Copper and iron disorders of the brain. Annu. Rev. Neurosci..

[48] De Bie P, Muller P, Wijmenga C, Klomp LWJ (2007). Molecular pathogenesis of Wilson and Menkes disease: Correlation of mutations with molecular defects and disease phenotypes. J. Med. Genet..

[49] Trist BG, Hare DJ, Double KL (2018). A proposed mechanism for neurodegeneration in movement disorders characterized by metal dyshomeostasis and oxidative stress. Cell Chem. Biol..

[50] Trist BG (2017). Amyotrophic lateral sclerosis-like superoxide dismutase 1 proteinopathy is associated with neuronal loss in Parkinson’s disease brain. Acta Neuropathol..

[51] Trist BG (2018). Accumulation of dysfunctional SOD1 protein in Parkinson’s disease is not associated with mutations in the SOD1 gene. Acta Neuropathol..

[52] Banci L (2010). Affinity gradients drive copper to cellular destinations. Nature.

[53] Bramer WM, Giustini D, de Jonge GB, Holland L, Bekhuis T (2016). De-duplication of database search results for systematic reviews in EndNote. J. Med. Libr. Assoc..

