# Supplementary material for: Empirical evidence for biometal dysregulation in Parkinson’s disease from a systematic review and Bradford Hill analysis
Source: NPJ Parkinsons Dis. 2022 Jun 27;8:83. doi: 10.1038/s41531-022-00345-4 (PMC9237090; doi:10.1038/s41531-022-00345-4)
Supplement: Supplementary file 1 — Supplementary Material [file 41531_2022_345_MOESM1_ESM.pdf]

Supplementary Material

Abdeen AH, Trist BG, Double KL (2022). *Empirical evidence for biometal dysregulation in Parkinson's disease: a systematic review and Bradford Hill analysis.*

Table of Contents

- *Supplementary Table 1: Outcome of the Bradford Hill analysis for each study* *2*
- *Supplementary Table 2: Overall results from application of the Bradford Hill model to studies investigating the etiological role of iron in Parkinson's disease* *20*
- *Supplementary Table 3: Overall results from application of the Bradford Hill model to studies investigating the etiological role of copper in Parkinson's disease* *21*
- *Supplementary Table 4: Comparison of Bradford Hill criteria outcomes for studies using atomic absorption spectroscopy or inductively coupled plasma–mass spectrometry* *22*
- *Supplementary Table 5: Bradford Hill analysis performed on iron and copper proteins/genes in Parkinson's disease* *23*
- *Supplementary Table 6: Literature search strategy for each database* *24*
- *Supplementary Table 7: Duplicate removal method* *24*
- *Supplementary Table 8: Inclusions and exclusion criteria for studies applied to the Bradford Hill model* *25*
- *Supplementary Table 9: Iron and copper proteins/genes investigated* *26*
- *Supplementary Table 10: Revised scoring rubric for the Genoud scale* *27*
- *References* 28

Study name	Author/Year	Investigation type	Quality	Bradford Hill Criterion	Outcome	Metal	Methods	Findings
Ceruloplasmin dysfunction and therapeutic potential for Parkinson disease	Ayton et al. 2013[1]	Post-mortem	Moderate	Strength & consistency	Supporting	Iron & copper	AAS	Reported a 42% increase and 50% reduction in PD SN iron and copper levels respectively, compared with age-matched controls.
				Specificity	Supporting	Iron & copper	AAS	No difference in cortical iron or copper metal levels between PD and control groups.
				Plausibility	Supporting	Copper	AAS	Reduced PD SN copper levels resulted in ceruloplasmin specific activity, which is associated with SN iron accumulation.
Neuromelanin associated redox-active iron is increased in the substantia nigra of patients with Parkinson's disease	Faucheux et al. 2003[2]	Post-mortem	Moderate	Strength & consistency	Supporting	Iron	Perl's stain	Non-heme iron (Fe(III)) binding to SN neuromelanin is increased in post-mortem PD brains compared with controls.
				Plausibility	Supporting	Iron	Perl's stain	Increased non-heme iron was correlated with significantly increased redox activity in the PD SNc, contributing to oxidative stress in this region
Iron and Aluminum Increase in the Substantia Nigra of Patients with Parkinson's Disease: An X-Ray Microanalysis	Hirsch et al. 1991[3]	Post-mortem	Moderate	Strength & consistency	Supporting	Iron	X-ray microanalysis	Relative assessment of metal levels showed a 3.4-fold increase of iron in PD SN zones lacking neuromelanin, compared with matched controls.
				Specificity	Supporting	Iron	X-ray microanalysis	Iron levels are unchanged in PD central gray substance compared with controls.
Increased Ndfip1 in the substantia nigra of Parkinsonian brains is associated with elevated iron levels	Howitt et al. 2014[4]	Post-mortem	High	Strength & consistency	Supporting	Iron	ICP-MS	Total iron concentrations measured in PD SN is significantly increased compared with control brains.
				Specificity	Supporting	Iron	ICP-MS	No difference in cortical iron metal levels between PD and control groups.
				Plausibility	Supporting	Iron	IHC	Elevated expression of Ndfip1 appears to be directly associated with the abnormal accumulation of a-synuclein in PD.
Brain iron and ferritin in Parkinson's and Alzheimer's diseases	Jellinger et al. 1990[5]	Post-mortem	Moderate	Strength & consistency	Supporting	Iron	Perl's stain	Semiquantitative assessment of Fe(III) showed a significant increase in PD SNc iron content.
				Plausibility	Supporting	Iron	Perl's stain	Iron accumulation was observed in active microglia, and was associated with microgliosis surrounding SN dopamine neurons, associated with microglial reactive oxygen species production and oxidative stress.
				Specificity	Equivocal	Iron	Perl's stain	Quantitation of biometal levels in PD frontal cortex and hippocampus demonstrated no change in iron levels compared with matched-controls. Iron accumulation was demonstrated in the SN of DLB patients.
Iron-Melanin Complex in Substantia Nigra of Parkinsonian Brains: An X-Ray Microanalysis	Jellinger et al. 1992[6]	Post-mortem	Moderate	Strength & consistency	Supporting	Iron	X-ray microanalysis	Iron content in neuromelanin of the PD SNc is elevated in comparison with controls.
Elemental micro-imaging and quantification of human substantia nigra using synchrotron radiation based x-ray fluorescence--in relation to Parkinson's disease	Szczerbowska-Boruchowska et al. 2012[7]	Post-mortem	Moderate	Strength & consistency	Supporting	Iron	SXFM	Alterations in intraneuronal and extraneuronal SN iron levels differentiate the PD and control group the most.
Regional metal concentrations in Parkinson's disease, other chronic neurological diseases, and control brains	Uitti et al. 1989[8]	Post-mortem	Moderate	Strength & consistency	Opposing	Iron	ICP-MS	No significant difference in SN metal concentrations were observed between PD and controls.
				Strength & consistency	Supporting	Copper	ICP-MS	Nigral copper was significantly reduced when three PD subgroups were compared with controls (p=0.0067).
				Specificity	Opposing	Iron	ICP-MS	No significant difference between SN metal concentrations and other regions in PD brain. No significant iron concentration differences were noted between brains from PD and other chronic neurologic disease.
				Specificity	Supporting	Copper	ICP-MS	Copper content in the PD SN was reduced compared with controls, though this was not seen in the frontal cortex, caudate nucleus and cerebellum.
	Visanji et al. 2013[9]	Post-mortem	Moderate	Strength & consistency	Equivocal	Iron	GF-AAS	No statistically significant increase in iron or ferritin in PD SN compared with control SN. However, increased ferroportin expression in the PD SN compared with control SN.

Iron deficiency in parkinsonism: Region-specific iron dysregulation in parkinson's disease and multiple system atrophy				Specificity	Equivocal	Iron	GF-AAS	Significant increase in iron in degenerating PD LC but not PD SNc. Iron accumulation was not found in SN of post-mortem MSA patients.
				Analogy	Supporting	Iron	WB	Iron levels and ferritin expression were increased, but ferroportin levels were decreased in MSA degenerating brain regions.
Iron and reactive oxygen species activity in parkinsonian substantia nigra	Wypijewska et al. 2010[10]	Post-mortem	High	Strength & consistency	Opposing	Copper	Mossbauer spectroscopy<br>Electrothermal AAS	No difference in nigral copper content was found in PD SN compared with healthy controls.
				Strength & consistency	Supporting	Iron	Mossbauer spectroscopy<br>Electrothermal AAS	An increase in the labile pool of iron was found in PD SN compared with control SN.
				Plausibility	Supporting	Iron	Electrothermal AAS<br>ROS assay	The concentration of labile iron is correlated with an increase in PD SN oxidative stress.
Transition metals, ferritin, glutathione, and ascorbic acid in parkinsonian brains	Riederer et al. 1989[11]	Post-mortem	Moderate	Biological gradient	Supporting	Copper	HPLC	The reduction in total glutathione levels per tissue weight was correlated with increasing neuropathological severity of PD.
				Strength & consistency	Opposing	Copper	AAS	Quantitation of biometal levels in PD SN demonstrated no change in copper levels compared with matched-controls.
				Strength & consistency	Supporting	Iron	AAS	Quantitation of biometal levels in the SN of advanced PD patients demonstrated increased iron(III) and total iron levels compared with matched-controls.
				Specificity	Supporting	Iron	AAS	Quantitation of biometal levels in 19 PD brains regions found iron accumulation was limited to the SN.
				Temporality	Opposing	Iron	AAS	Iron accumulation was identified in late-disease stage PD patients, but not in early-stage patients.
Individual dopaminergic neurons show raised iron levels in Parkinson disease	Oakley et al. 2007[12]	Post-mortem	Moderate	Strength & consistency	Supporting	Iron	Electron probe microanalysis	Neurons and neuropils demonstrated a significant increase in PD SN iron levels compared with controls.
				Specificity	Supporting	Iron	Electron probe microanalysis	Iron accumulation was not observed in the SN of Huntington's disease patients, who demonstrate loss in dopaminergic neurons.
				Biological gradient	Opposing	Iron	Electron probe microanalysis	No correlation was seen between neuronal iron levels and neuron counts in patients with PD and control subjects.
Complex I, iron, and ferritin in Parkinson's disease substantia nigra	Mann et al. 1994[13]	Post-mortem	Moderate	Strength & consistency	Supporting	Iron	ICP-MS	Iron levels in PD SN were elevated by 56% compared with controls.
				Plausibility	Opposing	Iron	ICP-MS<br>Enzyme activity	Complex I activity, used as an indicator for mitochondrial function, showed no correlation with iron concentrations in PD SN. Relative ferritin levels were not altered in response to changes in PD SN iron levels.
Transferrin and iron in normal, Alzheimer's disease, and Parkinson's disease brain regions	Loeffler et al. 1995[14]	Post-mortem	Moderate	Strength & consistency	Opposing	Iron	TIBC analyser	Iron concentrations were non-significantly altered in PD SN compared with both young and elderly control groups.
				Analogy	Equivocal	Iron	TIBC analyser	Iron accumulation is demonstrated in the degenerating frontal cortex, and non-degenerating globus pallidus of AD brains.
				Specificity	Opposing	Iron	TIBC analyser	Metal level analysis in AD brains demonstrated iron accumulation in the frontal cortex and globus pallidus, compared with young and elderly control groups.
				Biological gradient	Opposing	Iron	TIBC analyser	Transferrin expression and iron concentrations were more weakly correlated in PD SN, compared with elderly control tissues.
	Loeffler et al. 1996[15]	Post-mortem	High	Strength & consistency	Opposing	Copper	AAS	Quantitation of copper levels in the PD SN demonstrated no change compared with age-matched controls

Increased regional brain concentrations of ceruloplasmin in neurodegenerative disorders				Specificity	Opposing	Copper	AAS	Alterations in copper levels and ceruloplasmin protein expression were not restricted to the SN in PD, and was also presented in multiple brain regions in AD, HD and PSP.
				Analogy	Supporting	Copper	AAS	The degenerating SN of PSP patients demonstrated a 124% increase in ceruloplasmin levels compared with young controls.
Subcellular compartmentalisation of copper, iron, manganese, and zinc in the Parkinson's disease brain	Genoud et al. 2017[16]	Post-mortem	High	Strength & consistency	Supporting	Iron & copper	ICP-MS	Quantitation of biometal levels in the PD SN demonstrated a significant increase in iron levels, and a significant reduction in copper levels.
				Specificity	Supporting	Iron & copper	ICP-MS	Biometal distribution in the occipital cortex and fusiform gyrus was not altered in PD patients.
Distribution of iron in the basal ganglia and neocortex in postmortem tissue in Parkinson's disease and Alzheimer's disease	Griffiths et al. 1993[17]	Post-mortem	Moderate	Strength & consistency	Supporting	Iron	AAS	A two-fold increase in iron concentrations was observed in the PD SN, compared with healthy controls.
				Specificity	Opposing	Iron	AAS	The lateral globus pallidus, a non-degenerating region in the PD brain, demonstrated an elevation in brain iron levels.
Increased nigral iron content in postmortem Parkinsonian brain	Dexter et al. 1987[18]	Post-mortem	Moderate	Strength & consistency	Supporting	Iron & copper	ICP-MS	Metal levels in the PD SN showed a significant increase in iron levels, and a significant reduction in copper content.
				Specificity	Supporting	Iron & copper	ICP-MS	No significant changes were observed in cerebellar metal levels between PD and controls.
In vivo detection of iron and neuromelanin by transcranial sonography: A new approach for early detection of substantia nigra damage	Zecca et al. 2005[19]	Post-mortem	Moderate	Biological gradient	Supporting	Iron	AAS<br>ELISA	A significant positive correlation was found between SN echogenicity when plotted against iron concentration (r=0.298, P=0.006), H-ferritin (r=0.273, P=0.016) and L-ferritin (r=0.246, P=0.03).
				Temporality	Supporting	Iron	TCS	Two subjects with ILBD demonstrated significant increases in SN echogenicity compared with controls.
Iron levels in the human brain: A post-mortem study of anatomical region differences and age-related changes	Ramos et al. 2014[20]	Post-mortem	Moderate	Strength & consistency	Opposing	Iron	GF-AAS	There is a non-significant difference between SN iron levels between one PD, and control patients.
				Specificity	Opposing	Iron	GF-AAS	Iron accumulation was observed in caudate nucleus and hippocampus in AD, and the caudate nucleus and globus pallidus in PD.
				Analogy	Supporting	Iron	GF-AAS	Iron levels were increased in the hippocampus and caudate of AD patients compared with controls.
Biomonitorization of iron accumulation in the substantia nigra from Lewy body disease patients	Fernandez et al. 2017[21]	Post-mortem	Moderate	Specificity	Equivocal	Iron	AAS	Significantly increased iron levels were observed in the SN of LBD patients, but not in non-degenerating PD brain regions, or degenerating AD brain regions.
				Analogy	Supporting	Iron	AAS	Iron accumulation was identified in the degenerating SN of LBD patients compared with controls.
Copper pathology in vulnerable brain regions in Parkinson's disease	Davies et al. 2014[22]	Post-mortem	High	Strength & consistency	Supporting	Iron & copper	ICP-MS<br>SXFM<br>PIXE	Analysis of regional metal levels using ICP-MS demonstrated a significant difference in regional iron (+35%) and copper (-34%) content in comparison to age-matched controls. Additionally, iron and copper levels were significantly altered at the single cell level inside SN neurons (PIXE -- Fe +49%, Cu -65%; SXFM -- Fe +26%, Cu -65%).
				Specificity	Supporting	Iron & copper	SXFM<br>PIXE	Intracellular metal level analysis demonstrated a significant change in PD SN iron and copper levels, but not in the occipital cortex
				Temporality	Supporting	Copper	SXFM	SXFM analysis of post-mortem ILBD brains, considered to be a preclinical form of PD, demonstrated a 48% reduction in SN copper levels. No iron accumulation is observed in ILBD SN, in comparison with control groups.
				Biological gradient	Supporting	Copper	WB	A strong negative correlation was demonstrated between TH-associated CTR1 levels and disease duration (R^2=0.781, P=0.008).
				Plausibility	Supporting	Copper	ICP-MS, WB, SOD1 activity	Positive correlation between copper levels and SOD1 is consistent with the hypothesis that copper levels regulate SOD1 expression. Copper-dependent SOD1 activity is attenuated in the copper deficient SN, but is elevated in the anterior cingulate cortex where copper levels are normal.
Targeting chelatable iron as a therapeutic modality in Parkinson's disease	Devos et al. 2014[23]	Clinical	High	Experiment	Supporting	Iron	MRI R2*<br>UPDRS	Deferiprone—Phase II. Early stage Parkinson's disease patients quickly responded to treatment, as demonstrated by statistically significant improvements in SN iron levels and UPDRS motor indicators of disease progression over a 12-month period.

Ceruloplasmin activity and iron chelation treatment of patients with Parkinson's disease	Grolez et al. 2015[24]	Clinical	High	Experiment	Supporting	Iron	MRI R2*<br>UPDRS<br>CP activity	Deferiprone—Phase II. Patients displayed clinical and radiological improvement upon treatment, although patients with lower ceruloplasmin-ferroxidase activity respond more favourably to iron chelation therapy.
Brain iron chelation by deferiprone in a phase 2 randomised double-blinded placebo controlled clinical trial in Parkinson's disease	Martin-Bastida et al. 2017[25]	Clinical	High	Experiment	Opposing	Iron	MRI R2*<br>UPDRS	Deferiprone—Phase II. Iron chelation therapy for six month showed no significant improvements in UPDRS motor disability scores, or iron content within the SNc determined by R2 relaxation signal.
A quantitative analysis of isoferritins in select regions of aged, Parkinsonian, and Alzheimer's diseased brains	Connor et al. 1995[26]	Post-mortem	High	Strength & consistency	Supporting	Iron	WB	A significant reduction of H-ferritin and L-ferritin was observed in PD SN compared with healthy elderly controls.
				Specificity	Opposing	Iron	WB	Reduction in ferritin levels was observed in across multiple brain regions in PD (globus pallidus, frontal cortex) and in AD brains.
				Analogy	Supporting	Iron	Immunoassay	Isoferritin ratios were altered in the AD frontal cortex compared with healthy controls.
Decreased ferritin levels in brain in Parkinson's disease	Dexter et al. 1990[27]	Post-mortem	Moderate	Strength & consistency	Supporting	Iron	Rapid RIA	Patients who died with Parkinson's disease demonstrate markedly decreased (-48%) ferritin levels in the SNc compared with controls.
				Specificity	Opposing	Iron	Rapid RIA	In addition to the SNc, reduction in PD ferritin levels was reported in the cerebral cortex, putamen, globus pallidus and cerebellum.
Low Levels of Prohibitin in Substantia Nigra Makes Dopaminergic Neurons Vulnerable in Parkinson's Disease	Dutta et al. 2018[28]	Post-mortem	Moderate	Strength & consistency	Supporting	Iron	WB<br>Fluorescence microscopy	Whole tissue protein level of prohibitin is significantly decreased in the SN compared with controls. Immunolocalisation studies exhibit that the fluorescence intensity of prohibitin was significantly reduced in PD SN dopamine neruons compared with controls.
				Specificity	Opposing	Iron	WB<br>Fluorescence microscopy	Reduction in protein levels of prohibitin were reported in both the substantia nigra and ventral tegmental area.
The density of [125I]-transferrin binding sites on perikarya of melanized neurons of the substantia nigra is decreased in Parkinson's disease	Faucheux et al. 1997[29]	Post-mortem	Moderate	Strength & consistency	Supporting	Iron	Quantitative autoradiography	The density of transferrin receptors measured on perikarya of melanised neurons in the ventral SNc is significantly reduced in PD.
				Specificity	Supporting	Iron	Quantitative autoradiography	Reduction in transferrin receptor density is restricted to the ventral SNc, and does not extend to non-degenerating regions.
				Biological gradient	Supporting	Iron	Quantitative autoradiography	Changes in transferrin expression were most severe in the ventral tier of the SN compared with the dorsal tier in PD patients.
Distribution of 125I-ferrotransferrin binding sites in the mesencephalon of control subjects and patients with Parkinson's disease	Faucheux et al. 1993[30]	Post-mortem	Moderate	Strength & consistency	Opposing	Iron	Quantitative autoradiography	No significant difference in transferrin receptor expression was observed between PD SN and controls using quantitative autoradiography.
Lack of up-regulation of ferritin is associated with sustained iron regulatory protein-1 binding activity in the substantia nigra of patients with Parkinson's disease	Faucheux et al. 2002[31]	Post-mortem	Moderate	Plausibility	Opposing	Iron	WB<br>Electrophoretic mobility shift assays	Ferritin levels are not altered in the Parkinson's disease SN due to sustained IRP-1 binding activity to IRE.
Expression of lactoferrin receptors is increased in the mesencephalon of patients with Parkinson disease	Faucheux et al. 1995[32]	Post-mortem	Moderate	Strength & consistency	Supporting	Iron	Immunostaining	Stronger staining intensity of lactoferrin receptors was observed in dopaminergic regions affected in PD (ventral SNc +110%, dorsal SNc, +167%) compared with controls.
				Specificity	Supporting	Iron	Immunostaining	Although lactoferrin receptor protein expression was increased in non-vulnerable PD brain regions, the increase in the PD SN was statistically much greater than all other regions.
				Biological gradient	Supporting	Iron	Immunostaining	Lactoferrin receptor expression on ventrolateral SNc neurons was negatively correlated with dopaminergic neuron counts in PD patients (r=-0.67, P=0.07).

Mossbauer spectroscopy and ELISA studies reveal differences between Parkinson's disease and control substantia nigra	Galazka-Friedman et al. 2004[33]	Post-mortem	Moderate	Strength & consistency	Supporting	Iron	ELISA	The concentration of L-ferritin is signficiantly reduced in PD SN compared with healthy controls, whereas the overall ratio of H-ferritin to L-ferritin is increased.
Alterations in m-RNA expression for Cu,Zn-superoxide dismutase and glutathione peroxidase in the basal ganglia of MPTP-treated marmosets and patients with Parkinson's disease	Kunikowska & Jenner 2003[34]	Post-mortem	Moderate	Strength & consistency	Supporting	Copper	In situ hybdridisation histochemistry	mRNA expression of the copper proteins, SOD1 and glutathione peroxidase, were significantly reduced in post-mortem PD brains.
S-nitrosylation of divalent metal transporter 1 enhances iron uptake to mediate loss of dopaminergic neurons and motoric deficit	Liu et al. 2018[35]	Post-mortem	Moderate	Strength & consistency	Supporting	Iron	Resin-assisted capture assay	Expression of endogenous S-nitrosylation of DMT1 in post-mortem PD SN is markedly icnrease in comparison with controls.
Ceruloplasmin immunoreactivity in neurodegenerative disorders	Loeffler et al. 2001[36]	Post-mortem	Moderate	Strength & consistency	Opposing	Copper	Immunostaining	Ceruloplasmin reactivity was not changed in PD SN compared with healthy controls.
				Analogy	Supporting	Copper	Immunostaining	The expression of ceruloplasmin was increased in the hippocampus of AD patients compared with controls.
The absence of reactive astrocytosis is indicative of a unique inflammatory process in Parkinson's disease	Mirza et al. 1999[37]	Post-mortem	Moderate	Strength & consistency	Opposing	Copper	Immunostaining	There is no significant difference between immunoreactive glial cells labelled with metallothionein I/II in the PD SNc compared with controls.
				Strength & consistency	Supporting	Iron	Immunostaining	A higher density of ferritin-immunoreactive activated microglia was observed in the PD SN compared with the control SN.
				Plausibility	Opposing	Copper	Immunostaining	Elevated ferritin levels within microglia is associated with microglial activation and a neuroinflammatory phenotype.
				Specificity	Supporting	Iron	Immunostaining	No alterations in ferritin or metallothionein were observed in the non-degenerating putamen in PD compared with healthy controls.
Superoxide dismutase expression in Parkinson's disease	Poirier et al. 1994[38]	Post-mortem	Moderate	Strength & consistency	Supporting	Iron	Quantitative autoradiography	Levels of transferrin receptors were significantly reduced in the SN compared with controls.
				Specificity	Opposing	Iron	Quantitative autoradiography	In addition to PD SN, reduction in transferrin receptor expression was observed in the paranigral nucleus, red nucleus and oculomotor nucleus.
				Biological gradient	Opposing	Iron	Quantitative autoradiography	No correlation was observed between the density of transferrin binding sites and neuronal loss.
				Strength & consistency	Opposing	Copper	SOD1 activity assay	The activity of SOD1 in the PD SN is not significantly altered compared with controls.
A selective increase in particulate superoxide dismutase activity in parkinsonian substantia nigra	Saggu et al. 1989[39]	Post-mortem	Moderate	Strength & consistency	Opposing	Copper	SOD1 activity assay	The activity of cytosolic SOD1 is not altered between PD SN and controls.
Neural heme oxygenase-1 expression in idiopathic Parkinson's disease	Schipper et al. 1998[40]	Post-mortem	Moderate	Strength & consistency	Supporting	Iron	Immunostaining	Significant upregulation of HO-1 in PD SN astrocytes compared with age-matched controls in response to oxidative stress.
				Specificity	Supporting	Iron	Immunostaining	Percentage of astrocytes double-labelled with GFAP and HO-1 is significantly increased in the PD SN, but not other structures of the basal ganglia.
Amyotrophic lateral sclerosis-like superoxide dismutase 1 proteinopathy is associated with neuronal loss in Parkinson's disease brain	Trist et al. 2017[41]	Post-mortem	High	Strength & consistency	Supporting	Copper	IEF, WB, Immunostaining	The density of SOD1 aggregates was 8-fold higher in PD SNc compared with age-matched controls.
				Temporality	Supporting	Copper	Immunostaining	Misfolded SOD1 deposition was identified in the SN of ILBD patients, considered a prodromal form of PD, which was absent in matched-controls.
				Biological gradient	Supporting	Copper	IEF, WB, Immunostaining	SOD1 aggregate density was strongly inversely correlated with an index of neuronal loss (Pearson's $r = 0.903$, $p < 0.001$).
				Plausibility	Supporting	Copper	IEF, WB, Immunostaining	Changes in SOD1 protein expression, enzymatic activity, deposition and misfolding in the PD SN were proposed to result from neuronal copper deficiency in this brain region.

Accumulation of dysfunctional SOD1 protein in Parkinson's disease is not associated with mutations in the SOD1 gene	Trist et al. 2018[42]	Post-mortem	Moderate	Strength & consistency	Opposing	Copper	PCR	Impaired function of SOD1 protein in PD SN is not associated with mutations in the SOD1 gene.
Proteome analysis of human substantia nigra in Parkinson's disease	Werner et al. 2008[43]	Post-mortem	High	Strength & consistency	Equivocal	Iron & copper	2D-PAGE	H-ferritin, but not L-ferritin, is significantly increased in PD SN compared with control SN. Subtypes of glutathione transferase (M3, P1 & O1) are all significantly elevated in PD SN compared with control brains, though there are no differences in SOD1 and DJ-1.
Prevalence of haemochromatosis gene mutations in Parkinson's disease	Aamodt et al. 2007[44]	Clinical	Moderate	Strength & consistency	Opposing	Iron	PCR	Parkinson's disease is not associated with mutations in the haemochromatosis gene (C282Y, H63D and S65C).
Screening for mutations of the HFE gene in Parkinson's disease patients with hyperechogenicity of the substantia nigra	Akbas et al. 2006[45]	Clinical	High	Strength & consistency	Opposing	Iron	TCS<br>PCR	Parkinson's disease in patients previously characterised by increased SN iron content by TCS are not associated with HFE mutations (C282Y and H63D).
An association study between heme oxygenase-1 genetic variants and Parkinson's disease	Ayuso et al. 2014[46]	Clinical	High	Strength & consistency	Supporting	Iron	PCR	Two polymorphisms (VNTR (GT)n & SNP rs2071746) that modify the transcriptional activity of HO-1 gene are strongly associated with increased risk of developing PD.
				Temporality	Supporting	Iron	Genotyping	HO-1 gene mutations (VNTR (GT)n & SNP rs2071746) are strongly associated with increased risk of developing PD.
HFE gene mutations in a population of Italian Parkinson's disease patients	Biasiotto et al. 2008[47]	Clinical	High	Strength & consistency	Opposing	Iron	PCR	The most common HFE mutations (H63D & C282Y) are not associated with individual risk of developing PD.
				Biological gradient	Opposing	Iron	PCR	No significant variation in severity of symptoms on onset, and UPDRS scores of PD patients with no mutations, or one or more mutations.
Association study between iron-related genes polymorphisms and Parkinson's disease	Borie et al. 2002[48]	Clinical	Moderate	Strength & consistency	Equivocal	Iron	PCR	Analysis of intragenic polymorphisms of iron-related genes (transferrin receptor 1, HFE, lactoferrin) shows no difference between PD and controls. The G258S transferrin polymorphism, for which a higher frequency of the G allele was found among cases, particularly among cases with onset older than 60 and negative family history.
				Temporality	Supporting	Iron	Genotyping	The G258S transferrin mutation is present at a significantly higher frequency amongst cases of PD compared with controls.
The Cys282Tyr polymorphism in the HFE gene in Australian Parkinson's disease patients	Buchanan et al. 2002[49]	Clinical	High	Strength & consistency	Opposing	Iron	PCR-RFLP	Logistic regression of the HFE gene suggest that possession of the 282Tyr allele may reduce the risk of developing PD.
Screening of ferritin light polypeptide 460-461InsA mutation in Parkinson's disease patients in North America	Chen et al. 2002[50]	Clinical	Moderate	Strength & consistency	Opposing	Iron	PCR	Screening for an insertional (460-461InsA) mutation in the ferritin light polypeptide gene was not detected in 253 idiopathic PD patients.
Screening for mutations of the IRP2 gene in Parkinson's disease patients with hyperechogenicity of the substantia nigra	Deplazes et al. 2004[51]	Clinical	Moderate	Strength & consistency	Opposing	Iron	PCR	Genetic polymorphisms in the IRP2 gene (I88V, -88C>T, -74C>T) are not a common cause of PD associated with SN iron accumulation.
Genetic polymorphisms of superoxide dismutase in Parkinson's disease	Farin et al. 2001[52]	Clinical	Moderate	Strength & consistency	Opposing	Copper	PCR	No genetic variants were detected in the gene encoding SOD1 in DNA from idiopathic PD and control DNA extracts.
Screening for mutations of the ferritin light and heavy genes in Parkinson's disease patients with hyperechogenicity of the substantia nigra	Felletschin et al. 2003[53]	Clinical	Moderate	Strength & consistency	Opposing	Iron	PCR<br>DHPLC	Mutations in the ferritin light and heavy genes are not associated with SN iron accumulation in PD.

Analysis of nucleotide variations in genes of iron management in patients of Parkinson's disease and other movement disorders	Castigiloni et al. 2011[54]	Clinical	Moderate	Strength & consistency	Opposing	Iron & copper	PCR DHPLC	Analysis of nucleotide variations did not identify any sequence variations in CP, IRP2 or hepcidin genes that are associated with Parkinson's disease.
Analysis of ferritin genes in Parkinson disease	Foglieni et al. 2007[55]	Clinical	Moderate	Strength & consistency	Opposing	Iron	PCR DHPLC	Mutations in ferritin genes are not commonly associated with PD.
Genetic analysis of heme oxygenase-1 (HO-1) in German Parkinson's disease patients	Funke et al. 2009[56]	Clinical	High	Strength & consistency	Opposing	Iron	SNaPshot analysis qRT-PCR	Genetic analysis of HO-1 gene for (GT)n fragment length and three coding SNPs showed no association between these genetic markers and PD.
Genetic linkage studies in autosomal dominant parkinsonism: Evaluation of seven candidate genes	Gasser et al. 1994[57]g	Clinical	High	Strength & consistency	Opposing	Iron & copper	PCR	Summed lod scores for three families with inherited parkinsonism excluded linkage to the genes glutathione peroxidase 1, TH, SOD1 and catalase
Association study between four polymorphisms in the HFE, TF and TFR genes and Parkinson's disease in Southern Italy	Greco et al. 2011[58]	Clinical	Moderate	Strength & consistency	Opposing	Iron	PCR	Genetic polymorphisms in HFE and TFR genes demonstrate no differences in genotype and allele frequencies between PD and control groups.
Association of HFE common mutations with Parkinson's disease, Alzheimer's disease and mild cognitive impairment in a Portuguese cohort	Guerreiro et al. 2006[59]	Clinical	High	Biological gradient	Opposing	Iron	PCR	There are no statistically significant differences in the age of onset of PD for wildtype, heterozygous and homozygous HFE mutations (H63D and C282Y).
				Temporality	Supporting	Iron	PCR	The presence of the HFE C282Y variant allele may confer higher risk for developing PD.
DMT1 polymorphism and risk of Parkinson's disease	He et al. 2011[60]	Clinical	High	Strength & consistency	Opposing	Iron	PCR-RFLP	A haplotype of DMT1 (C alleles of 1254T and IVS+44C/A polymorphisms) occurred at greater frequencies in PD compared with controls, although there was no significant association between this haplotype and PD.
Ceruloplasmin gene variations and substantia nigra hyperechogenicity in Parkinson disease	Hochstrasser et al. 2005[61]	Clinical	High	Strength & consistency	Supporting	Copper	TCS DHPLC	Sequence variations of the ceruloplasmin genes (9/D544E; 13/R793H) are associated with both PD, and SN hyperechogencity.
Polymorphisms in iron-responsive binding protein 2 and lack of association with sporadic Parkinson's disease	Lee et al. 2002[62]	Clinical	Moderate	Strength & consistency	Opposing	Iron	PCR	IRP2 genetic polymorphisms (L159V, F272L and T560I) lack an association with idiopathic PD.
Association between sex, systemic iron variation and probability of Parkinson's disease	Mariani et al. 2016[63]	Clinical	High	Strength & consistency	Opposing	Iron	PCR	Genetic screening of PD patients reveals no association between HFE (C282Y, H63D) and TF (P589S) mutations, and increased PD risk.
Effects of hemochromatosis and transferrin gene mutations on peripheral iron dyshomeostasis in mild cognitive impairment and Alzheimer's and Parkinson's diseases	Mariani et al. 2013[64]	Clinical	High	Strength & consistency	Opposing	Iron & copper	PCR	Genetic investigation of transferrin (P589S), HFE (H63D and C282Y) and ceruloplasmin (D544E, R793H) showed no differences in allelic and genotype distributions between PD patients and controls.
Absence of mutations in superoxide dismutase and catalase genes in patients with Parkinson's disease	Parboosingh et al. 1995[65]	Clinical	Moderate	Strength & consistency	Opposing	Iron & copper	PCR	No SOD1 or catalase gene mutations were identified in PD patients.
Pooled analysis of iron-related genes in Parkinson's disease: Association with transferrin	Rhodes et al. 2014[66]	Clinical	High	Strength & consistency	Opposing	Iron	PCR	Protective association between PD and a haplotype composed of the G allele at rs10247962 and the A allele at rs4434553 in the transferrin receptor 2 gene.
				Temporality	Supporting	Iron	PCR	A protective association was identified between transferrin and transferrin receptor 2 alleles and risk of PD, according to varying gene allele frequencies between PD and control groups.

Mutations in the hemochromatosis gene (HFE), Parkinson's disease and parkinsonism	Dekker et al. 2003[67]	Clinical	Moderate	Strength & consistency	Supporting	Iron	PCR	PD patients were significantly more often homozygous for the HFE C282Y mutation than controls (P=0.03).
				Analogy	Supporting	Iron	PCR	Patients with non-PD parkinsonism showed a significant increase in homozygous HFE C282Y mutations compared with controls.
Is the 1254T>C polymorphism in the DMT1 gene associated with Parkinson's disease?	Saadat et al. 2015[68]	Clinical	Moderate	Strength & consistency	Supporting	Iron	PCR	The T allele, and TT genotype of the 1254T>C polymorphism in the DMT1 gene were associated with PD.
				Temporality	Supporting	Iron	Genotyping	An association was identified between DMT1 SNP 1254T>C (homozygous and heterozygous) and increased PD risk.
Association between a heme oxygenase-2 genetic variant and risk of Parkinson's disease in Han Chinese	Tian et al. 2017[69]	Clinical	High	Strength & consistency	Opposing	Iron	PCR	Haplotype analysis showed that SNP rs1051308 in HO-1 is associated with risk of PD in Han Chinese.
Indices of oxidative stress and mitochondrial function in individuals with incidental Lewy body disease	Dexter et al. 1994[70]	Post-mortem	Moderate	Temporality	Opposing	Iron & copper	ICP-MS HPLC-UV	SN iron, copper and glutathione levels were not significantly altered in ILBD patients compared with controls.
Single-nucleotide polymorphisms and haplotypes of non-coding area in the CP gene are correlated with Parkinson's disease	Zhao et al. 2015[71]	Clinical	High	Strength & consistency	Supporting	Copper	PCR Microarray analysis	Common genetic variants of ceruloplasmin are associated with Parkinson's disease, but not with control subjects.
				Temporality	Supporting	Iron & copper	Genotyping	The frequencies of eight SNPs of ceruloplasmin, and their haplotypes were significantly different between PD patients and controls.
				Biological gradient	Opposing	Copper	PCR Microarray analysis	The frequencies of the ceruloplasmin gene SNPs are not associated with Hoehn and Yahr staging in PD patients.
The whole-brain pattern of magnetic susceptibility perturbations in Parkinson's disease	Acosta-Cabronero et al. 2017[72]	Clinical	High	Strength & consistency	Supporting	Iron	QSM	Visualisation of nigrosome-1 via QSM imaging demonstrates iron overload (+17%) in PD patients compared with controls.
				Specificity	Opposing	Iron	QSM	Age-corrected QSM statistics demonstrate elevated iron levels in the lateral occipital, middle temporal, posterior parietal and rostral middle prefrontal lobes.
				Biological gradient	Opposing	Iron	QSM	No correlation was observed between SN mean susceptibility values and UPDRS motor scores or MMSE scores.
Quantifying iron deposition within the substantia nigra of Parkinson's disease by quantitative susceptibility mapping	An et al. 2018[73]	Clinical	Moderate	Strength & consistency	Supporting	Iron	QSM	Patients at different stages of PD (mild-severity and advanced-severity), and with different disease subtypes (tremor dominant, akinetic/rigidity dominant, and mixed) showed a significant increase in the susceptibility of the SN in comparison with healthy controls.
				Biological gradient	Supporting	Iron	QSM	Magnetic susceptibility of SN, an indicator of regional iron levels, is correlated significantly with Hoehn-Yahr stages (r=0.417; p=0.005) and UPDRS (r =0.300; p=0.048).
T2 relaxation time in patients with Parkinson's disease	Antonini et al. 1993[74]	Clinical	Moderate	Strength & consistency	Supporting	Iron	T2	Significant shortening of the T2 signal was found in the PD patient group suggesting regional iron deposition.
				Specificity	Opposing	Iron	T2	Significant shortening of T2 signal was demonstrated in the caudate nucleus and total putamen of PD patients.
				Biological gradient	Opposing	Iron	T2	MRI T2 relaxation times were not correlated with disease duration or clinical severity of PD.
Brain iron concentrations in regions of interest and relation with serum iron levels in Parkinson disease	Costa-Mallen et al. 2017[75]	Clinical	High	Strength & consistency	Supporting	Iron	T2'	MRI measurements showed a significant increase in PD SNc R2' relaxation times compared with age- and sex-matched controls
				Specificity	Supporting	Iron	T2'	Investigated regions of interest of PD patients, including the red nucleus, globus pallidus, white matter and putamen, did not show a significant change in R2' times compared with controls.
Substantia nigra in Parkinson's disease: A multimodal MRI comparison between early and advanced stages of the disease	Aquino et al. 2014[76]	Clinical	Moderate	Strength & consistency	Opposing	Iron	R2*	The R2* values in the PD SN were not significantly changed in comparison with control subjects.
				Biological gradient	Opposing	Iron	R2*	MRI R2* values were not significantly altered between early and advanced PD disease stage patients, who were assessed by UPDRS scores.

Substantia nigra locations of iron-content, free-water and mean diffusivity abnormalities in moderate stage Parkinson's disease	Arribarat et al. 2019[77]	Clinical	High	Strength & consistency	Supporting	Iron	R2*	R2* relaxation time in the anterior SN, but not the posterior SN, was significantly increased in PD patients compared with age-matched controls, indicating iron accumulation in PD patients.
				Biological gradient	Opposing	Iron	R2*	MRI R2* values in the SN were not correlated with clinical score changes from baseline to a two-year follow-up time point.
T2-weighted MRI in Parkinson's disease; substantia nigra pars compacta hypointensity correlates with the clinical scores	Atasoy et al. 2004[78]	Clinical	High	Strength & consistency	Supporting	Iron	T2	T2 data demonstrated increased iron levels in the SNc of PD patients compared with controls.
				Specificity	Opposing	Iron	T2	Iron accumulation was demonstrated in the dentate nucleus of PD patients, when compared with healthy control subjects.
				Biological gradient	Supporting	Iron	T2	SNc T2-intensity had a significant negative correlation with UPDRS (r=0.625; p=0.003) and rigidity (r=0.503; p=0.024) scores in PD patients.
Lateral asymmetry and spatial difference of iron deposition in the substantia nigra of patients with Parkinson disease measured with quantitative susceptibility mapping	Azuma et al. 2016[79]	Clinical	High	Strength & consistency	Supporting	Iron	QSM	Mean susceptibility value was significantly higher in the posterior SN of the more affected hemibrain, compared with the less affected hemibrain, as well as healthy controls.
				Specificity	Supporting	Iron	QSM	Both the most and less affected hemibrains of PD patients demonstrated elevation in susceptiblity values that were restricted to different SN compartments, and not other brain regions.
Quantifying brain iron deposition in patients with Parkinson's disease using quantitative susceptibility mapping, R2 and R2	Barbosa et al. 2015[80]	Clinical	High	Strength & consistency	Supporting	Iron	R2, R2*, QSM	R2, R2* and QSM maps detected a signal change indicating iron accumulation in PD SN patients, compared with healthy controls.
				Specificity	Supporting	Iron	R2, R2*, QSM	Data from all three MRI techniques demonstrated that iron accumulation is specific to the SN or SNc, and does not extend to other structures of the basal ganglia.
MRI evaluation of brain iron in earlier- and later-onset Parkinson's disease and normal subjects	Bartzokis et al. 1999[81]	Clinical	High	Strength & consistency	Equivocal	Iron	FDRI, R2*	FDRI values suggest increased ferritin-bound iron in the SNc of early-onset PD patients, but not late-stage PD patients. No change in free iron was observed in early or late stage PD patients compared with controls.
				Specificity	Opposing	Iron	FDRI, R2*	FDRI values were also increased in putamen, globus pallidus and white matter of early-stage PD patients.
				Biological gradient	Equivocal	Iron	FDRI, R2*	Reduction in FDRI values between early and late onset patients suggests release of iron from ferritin as disease progresses to elevate free iron, however free iron levels were not elevated as the disease progressed according to low-field R2 values.
				Strength & consistency	Equivocal	Iron	FDRI, R2*	FDRI values suggest increased ferritin-bound iron in the SNc of early-onset PD patients, but not late-stage PD patients. No change in free iron was observed in early or late stage PD patients compared with controls
				Specificity	Opposing	Iron	FDRI, R2*	FDRI values were also increased in putamen, globus pallidus and white matter of early-stage PD patients
				Biological gradient	Equivocal	Iron	FDRI, R2*	Reductions in FDRI values between early and late onset patients suggests release of iron from ferritin as disease progresses to elevate free iron, however free iron levels were not elevated as the disease progressed according to low-field R2 values
Quantitative mapping of T1 and T2 discloses nigral and brainstem pathology in early Parkinson's disease	Baudrexel et al. 2010[82]	Clinical	Moderate	Strength & consistency	Supporting	Iron	T1, T2*	T1 and T2* relaxation times are decreased in the rostral PD SN indicating neuronal loss and regional iron deposition.
				Biological gradient	Opposing	Iron	T2*	No correlation was observed between caudal and rostral nigral PD T2* values and UPDRS III.
Hyperechogenicity of the substantia nigra in healthy controls is related to MRI changes and to neuronal loss as determined by F-Dopa PET	Behnke et al. 2009[83]	Clinical	Moderate	Strength & consistency	Supporting	Iron	TCS<br>T2	T2 relaxation times are significnatly reduced in PD patients characterised by a hyperechogenic SN, compared with healthy controls lacking hyperechogenicity.
				Temporality	Supporting	Iron	TCS<br>T2	Iron accumulation in healthy controls is associated with SN structural change and nigrostriatal dysfunction are hypothesised to precede PD.
Ventral posterior substantia nigra iron increases over 3 years in Parkinson's disease	Bergsland et al. 2019[84]	Clinical	Moderate	Strength & consistency	Supporting	Iron	QSM	Magnetic susceptibility measured over three years in PD patients showed a significant, longitudinal increase in the ventral posterior SN, which corresponds to an annual increase of 3.5%.

				Biological gradient	Equivocal	Iron	QSM	No significant associations were detected between any baseline SN magnetic susceptibility assessment and baseline UPDRS III (P=0.319) or Hoeh-Yahr (P=0.305) scores. Higher QSM values were identified in the ventral, but not dorsal, tier of the PD SN compared with the control SN.
Motor phenotype and magnetic resonance measures of basal ganglia iron levels in Parkinson's disease	Bunzeck et al. 2013[85]	Clinical	High	Strength & consistency	Opposing	Iron	R2*	R2* relaxation rate of PD SN showed a trend to significance (P=0.09) compared with healthy controls.
				Biological gradient	Supporting	Iron	R2*	There was an inverse correlation between magnetization transfer and R2* values in the SN, indicating greater neurodegeneration with increased iron accumulation.
Iron deposition in Parkinson's disease by quantitative susceptibility mapping	Chen et al. 2019[86]	Clinical	Moderate	Strength & consistency	Supporting	Iron	QSM	A significant increase in PD SN susceptibility values was observed compared with healthy volunteers.
				Biological gradient	Supporting	Iron	QSM	A significant increase in SN susceptibility values was observed in late PD patients (H-Y stage >3) and early PD patients (H-Y stage <2.5).
				Specificity	Opposing	Iron	QSM	In addition to the SNc, the red nucleus showed elevated susceptibility values in late PD patients compared with early PD patients.
Iron accumulation is not homogenous among patients with Parkinson's disease	Dashtipour et al. 2015[87]	Clinical	High	Strength & consistency	Opposing	Iron	SWI	Average iron levels measured by susceptibility weighted imaging were not significant in different ROIs in PD patients compared with control subjects.
Serum cholesterol and nigrostriatal R2* values in Parkinson's disease	Du et al. 2012[88]	Clinical	High	Strength & consistency	Supporting	Iron	R2*	The R2* values in the SN of PD subjects were significantly higher compared with that of controls.
				Specificity	Supporting	Iron	R2*	R2* values were not altered in the caudate, putamen and globus pallidus in PD compared with controls.
				Biological gradient	Supporting	Iron	R2*	SN R2* values are correlated with indicators of disease severity, including UPDRS-III motor scores (r=0.355, p=0.039), and disease duration (r=0.418, p=0.014).
Combined R2* and Diffusion Tensor Imaging Changes in the Substantia Nigra in Parkinson's Disease	Du et al. 2011[89]	Clinical	High	Biological gradient	Opposing	Iron	R2*	No significant correlations between either UPDRS-III scores and R2* relaxation times (r^2 = 0.2686, P=0.3145), or between disease duration and R2* relaxation times (r^2 = -0.0712, P=0.9747)
				Strength & consistency	Supporting	Iron	R2*	PD subjects had significant increase in bilateral R2* values in the SN (P<0.0001).
Quantitative susceptibility mapping of the midbrain in Parkinson's disease	Du et al. 2016[90]	Clinical	Moderate	Strength & consistency	Supporting	Iron	R2* QSM	QSM and R2* values both show a significant increase in SNc PD compared with healthy controls.
				Biological gradient	Equivocal	Iron	R2* QSM	QSM values in the SNc were highly correlated with disease duration (r=0.423, P=0.003) and UPDRS-II (r=0.345, P=0.018). R2* values are not correlated with neither disease duration nor UPDRS I & III scores.
Potential of diffusion tensor imaging and relaxometry for the detection of specific pathological alterations in Parkinson's Disease (PD)	Esterhammer et al. 2015[91]	Clinical	Moderate	Strength & consistency	Supporting	Iron	R2, R2*	R2 and R2* values show a significant increase between healthy controls and PD patients with a disease duration < 5, and between PD patients with a disease duration of <5 and >5.
				Specificity	Supporting	Iron	R2, R2*	R2 and R2* values are signifciantly increased in the PD SN, but not corpus callosum, thalamus, globus pallidus, putamen and caudate nucleus.
				Biological gradient	Opposing	Iron	R2, R2*	No signficiant correlations between MRI relaxation times in the SN and UPDRS-III scores or Hoehn-Yahr staging were observed.
Regional high iron in the substantia nigra differentiates Parkinson's disease patients from healthy controls	Ghassaban et al. 2019[92]	Clinical	High	Strength & consistency	Supporting	Iron	R2* QSM	The SN showed significantly higher susceptibility values in PD patients when compared with the healthy control cohort.
				Specificity	Supporting	Iron	R2* QSM	Susceptibility mean and R2* values were significantly increased in the PD SN, but not the caudate nucleus, globus pallidus, putamen, thalamus, red nucleus and dentate nucleus.
				Biological gradient	Supporting	Iron	QSM	Dividing the PD cohort in two sub-groups with normal and elevated susceptibility values, showed that UPDRS-III scores are significantly increased in patients with greater iron content.
Increased iron-related MRI contrast in the substantia nigra in Parkinson's disease	Gorell et al. 1995[93]	Clinical	High	Strength & consistency	Supporting	Iron	R2, R2*, R2'	Average SN values showed that PD subjects had significantly lower values of R2 (P=0.046), as well as higher values of R2* (p=0.001) and R2' (p<0.001) compared with controls.

Brain iron deposition in Parkinson's disease imaged using the PRIME magnetic resonance sequence	Graham et al. 2000[94]	Clinical	High	Biological gradient	Equivocal	Iron	R2, R2*, R2'	The asymmetry of short reaction time performance was not significantly correlated with asymmetry of R2, although strongly correlated with asymmetries of R2* (r=0.91, P=0.001) and R2' (r=0.72, P=0.03).
				Strength & consistency	Supporting	Iron	R2, R2*, R2'	Mean relaxation rates showed that R2* and R2' are significantly higher in PD compared with healthy controls, but no change was observed for R2.
				Specificity	Supporting	Iron	R2*, R2'	MRI R2* and R2' data demonstrated that iron accumulation that is restricted to the PD SN.
				Biological gradient	Opposing	Iron	R2, R2*, R2'	No correlation between R2, R2*, R2' relaxation rates of PD SN and disease duration or UPDRS scores.
Regionally progressive accumulation of iron in Parkinson's disease as measured by quantitative susceptibility mapping	Guan et al. 2015[95]	Clinical	High	Strength & consistency	Supporting	Iron	R2* QSM	R2* relaxation times and mean susceptibility values are increased in the early and late PD SNc compared with matched controls.
				Specificity	Equivocal	Iron	R2* QSM	R2* relaxation rate is only increased in the PD SN, whereas mean susceptibility values were significantly increased in the SNc, SNr and globus pallidus in late PD compared with controls.
				Biological gradient	Supporting	Iron	QSM	QSM values in the PD SNc were correlated with the Hoehn-Yahr stage (r=0.375; p=0.004) and the UPDRS motor scores (r=0.335; p=0.010).
Influence of regional iron on the motor impairments of Parkinson's disease: A quantitative susceptibility mapping study	Guan et al. 2017[96]	Clinical	Moderate	Strength & consistency	Supporting	Iron	R2* QSM	Magnetic susceptibility and R2* relaxation values indicated a significant increase in iron levels in tremor-dominant PD and akinetic/rigidity-dominant PD compared with healthy controls.
				Specificity	Equivocal	Iron	R2* QSM	Magnetic susceptibility values are increased in the SNc, red nucleus and dentate nucleus in tremor-dominant PD, however R2* values were only increased in the SNc.
				Biological gradient	Supporting	Iron	R2* QSM	For total motor impairment, increasing UPDRS motor scores (r=0.419, P=0.001) and Hoehn-Yahr stages (r=0.373, P=0.004) were significantly associated with increasing SNc magnetic susceptiblity.
Iron-related nigral degeneration influences functional topology mediated by striatal dysfunction in Parkinson's disease	Guan et al. 2019[97]	Clinical	Moderate	Strength & consistency	Supporting	Iron	QSM	Significant iron accumulation was observed in the inferior SNc of PD patients compared with normal controls.
				Biological gradient	Supporting	Iron	QSM	Iron content in the inferior SNc was significantly correlated with PDQ-39 score (r=0.484, P < 0.001).
				Plausibility	Supporting	Iron	QSM	Iron accumulation observed in PD Patients was associated with disrupted functional striatal interconnectivitiy between the two hemispheres.
Region-specific disturbed iron distribution in early idiopathic Parkinson's disease measured by quantitative susceptibility mapping	He et al. 2015[98]	Clinical	Moderate	Strength & consistency	Supporting	Iron	R2* QSM	Early-stage PD patients showed an increase in SN susceptibility and R2* values, compared with healthy controls. However, Hoehn-Yahr Stage I PD patients exhibit an increase in bilateral SN susceptibility, but not R2*, values.
				Specificity	Equivocal	Iron	QSM	Early-stage PD patients showed a specific increase in SN susceptibility values. Hoehn-Yahr Stage I PD patients show an increase in contralateral putamen magnetic susceptibility values.
				Biological gradient	Supporting	Iron	QSM	Regression analysis showed a significant relationship between susceptibility values in bilateral SN and clinical measures, including disease duration and UPDRS III, in early-stage PD.
Nigral iron deposition in common tremor disorders	Homayoon et al. 2019[99]	Clinical	High	Strength & consistency	Supporting	Iron	R2*	Significant higher R2* values in SN were found in tremor-dominant PD when compared with tremor in dystonia, essential tremor and control groups (P<0.023).
				Biological gradient	Supporting	Iron	R2*	Nigral PD R2* relaxation rates showed a significant positive correlation with disease duration (r=0.369, P=0.019) and MDS-UPDRS scores (r=0.346, p=0.031).
Magnetic resonance imaging features of the nigrostriatal system: Biomarkers of Parkinson's disease stages?	Hopes et al. 2016[100]	Clinical	High	Strength & consistency	Supporting	Iron	R2*	A significant increase in SN iron overload determined by the R2* value is seen in de novo, early-stage and advanced-stage PD patients.
				Specificity	Opposing	Iron	R2*	Radiological data show a significant increase in R2* values in the caudate nucleus and putamen of de novo, early-stage and advanced-stage PD compared with controls.

				Biological gradient	Supporting	Iron	R2*	Longitudinal analysis in 35 early-stage PD patients showed a statistically significant correlation between the change in R2* in the SN and the changes in the UPDRS motor scores (r^2=-0.5; p=0.03).
Transcranial imaging of substantia nigra hyperechogenicity in a Taiwanese cohort of Parkinson's disease	Huang et al. 2007[101]	Clinical	High	Strength & consistency	Supporting	Iron	R2*	SN hyperechogenicity and a higher hyper-SN/ipsilateral midbrain ratio were observed in early and late onset PD patients compared with the SN of healthy controls.
Quantitative assessment of iron deposition in Parkinson's disease using enhanced T2 star-weighted angiography	Ji et al. 2016[102]	Clinical	High	Strength & consistency	Supporting	Iron	R2*	A significant increase in R2* signal values was observed in PD SN compared with controls.
				Specificity	Opposing	Iron	R2*	The relaxation rate R2* was increased in the red nuclei bilaterally in PD patients compared with controls.
				Biological gradient	Equivocal	Iron	R2*	No correlation was observed between R2* signal in SN PD patients and UPDRS-III scores. Signal values of the SN of patients with a Hoehn-Yahr score >1 were slightly correlated with UPDRS-III scores.
Nigral iron deposition occurs across motor phenotypes of Parkinson's disease	Jin et al. 2012[103]	Clinical	High	Strength & consistency	Supporting	Iron	SWI	Bilateral SN phase values were significantly reduced in non-medicated PD patients compared with healthy volunteers (p=0.011).
				Specificity	Supporting	Iron	SWI	Significant differences in phase values of brain regions outside of the SN were not altered compared with controls (p>0.05).
				Biological gradient	Equivocal	Iron	SWI	Hoehn-Yahr staging correlated with the bilateral average phase values of the PD SN. UPDRS motor scores were correlated with SN phase values in akinetic/rigidity PD (r=-0.330, P=0.027), but not in tremor-dominant PD (r=-0.003, P=0.991) or PD with mixed symptomology (r=-0.040, P=0.907).
Decreased serum ceruloplasmin levels characteristically aggravate nigral iron deposition in Parkinson's disease	Jin et al. 2011[104]	Clinical	High	Strength & consistency	Supporting	Iron	SWI	PD patients exhibited a decline in nigral bilateral average phase values compared with control subjects.
				Specificity	Supporting	Iron	SWI	With the exception of the SN, the bilateral average phase values of all other brain regions measured did not differ significantly between PD and controls.
				Biological gradient	Supporting	Iron	SWI	A significant correlation was observed between nigral bilateral average phase values and UPDRS motor score (r=-0.369, P=0.013).
Serum Uric acid and nigral iron deposition in Parkinson's disease: A pilot study	Kim & Lee 2014[105]	Clinical	Moderate	Strength & consistency	Supporting	Iron	SWI	PD SN phase radians were significantly reduced in comparison to age-matched controls.
				Specificity	Supporting	Iron	SWI	Reduction in phase values was observed in the SN only, and not other structure of the basal ganglia.
				Biological gradient	Opposing	Iron	SWI	Phase shift values in PD SN demonstrated no significant correlations with UPDRS-III scores and disease duration.
MRI evaluation of the basal ganglia size and iron content in patients with Parkinson's disease	Kosta et al. 2006[106]	Clinical	High	Strength & consistency	Supporting	Iron	T2	A significant decrease in T2 relaxation time was observed in PD SNc (bilaterally and in the more affected side) compared with controls.
				Specificity	Supporting	Iron	T2	The SNc was the only brain region that demonstrated a significant reduction in T2 relaxation time in PD patients.
Diffusion tensor imaging of the substantia nigra in Parkinson's disease revisited	Langley et al. 2017[107]	Clinical	Moderate	Strength & consistency	Supporting	Iron	T2*	The lateral ventral SNc showed a significant increase in T2*-weighted signal hypointensity compared with controls.
				Biological gradient	Supporting	Iron	T2*	No correlation was observed between PD SN T2* hypointensity and UPDRS-III score (R^2=0.0005, P=0.964) or disease duration (P=0.149, R^2=0.45). Phase shifts were much higher in the lateral ventral SN in PD patients; this is the nigral sub-region within which the most severe nigral degeneration occurs.
Higher iron in the red nucleus marks Parkinson's dyskinesia	Lewis et al. 2013[108]	Clinical	High	Strength & consistency	Supporting	Iron	R2*	PD subjects, with and without dyskinesia, demonstrated significantly increased R2* values in the SN.
				Specificity	Equivocal	Iron	R2*	A significant increase in R2* signal value was observed in the red nucleus of PD patients with dyskinesia, but not in patients without dyskinesia.
				Biological gradient	Supporting	Iron	R2*	R2* relaxation rate values recorded in PD patients showed a significant correlation with UPDRS-III (r=0.364, P=0.025) and disease duration (r=0.357, P=0.028).

Iron deposition in substantia nigra: abnormal iron metabolism, neuroinflammatory mechanism and clinical relevance	Liu et al. 2017[109]	Clinical	High	Strength & consistency	Supporting	Iron	SWI	Corrected phase values in the PD SN are significantly reduced compared with controls.
				Specificity	Supporting	Iron	SWI	No significant change in phase values was observed in PD SN compared with controls.
				Biological gradient	Supporting	Iron	SWI	Iron deposition in PD patients measured via SWI was correlated with motor and non-motor decline.
				Plausibility	Supporting	Iron	SWI	Corrected phase values of the SN in PD patients were negatively correlated with the concentration of interleukin-1β in CSF, hypothesised to be a result of excess SN iron.
High resolution magnetic susceptibility mapping of the substantia nigra in Parkinson's disease	Loftipour et al. 2012[110]	Clinical	High	Strength & consistency	Supporting	Iron	T2*	A significant difference in SNc T2* values was demonstrated between PD patients and control subjects (P=0.042).
Midbrain iron content in early Parkinson disease: A potential biomarker of disease status	Martin et al. 2008[111]	Clinical	High	Strength & consistency	Supporting	Iron	R2*	Transverse relaxation rate R2* was elevated by 18% in the lateral SNc compared with age and sex-matched controls.
				Specificity	Supporting	Iron	R2*	A significant change in the tranverse relaxation rate R2* between PD and controls was specific to the lateral SNc, and was not altered in the SNr, globus pallidus, putamen, caudate nucleus, red nucleus and anterior white matter.
				Biological gradient	Supporting	Iron	R2*	There was an association between the R2* relaxation values for the lateral SNc from the most affected brain side and contralateral UPDRS motor scores (r^2=0.27, P<0.01).
Motor associations of iron accumulation in deep grey matter nuclei in Parkinson's disease: a cross-sectional study of iron-related magnetic resonance imaging susceptibility	Martin-Bastida et al. 2017[112]	Clinical	High	Strength & consistency	Supporting	Iron	SWI	Patients with different levels of PD severity, assessed by UPDRS, revealed a significant difference in SN radians compared with age-matched controls. Higher UPDRS scores were associated with a greater level of significance.
				Specificity	Opposing	Iron	SWI	Putaminal and pallidal radians were significantly higher in patients with different levels of PD severity compared with controls. Only putaminal radians in PD group 3 did not show significance.
				Biological gradient	Supporting	Iron	SWI	Pearson's correlation analysis revealed significant positive correlations between SN radians and total UPDRS-III (r=0.420, P<0.001) and bradykinesia-rigidity subscores (r=0.407, P=0.001), and a trend to significance for tremor (r=0.219, P=0.071).
Plasma ceruloplasmin ferroxidase activity correlates with the nigral sonographic area in Parkinson's disease patients: A pilot study	Martinez-Hernandez et al. 2011[113]	Clinical	High	Strength & consistency	Supporting	Iron	TCS	PD patients demonstrate a significant increase in SN echogenicity compared with healthy volunteers.
Topographical differences of brain iron deposition between progressive supranuclear palsy and parkinsonian variant multiple system atrophy	Han et al. 2013[114]	Clinical	High	Strength & consistency	Supporting	Iron	SWI	A significant increase in phase shift values was observed in PD SN compared with controls.
				Specificity	Equivocal	Iron	SWI	Iron accumulation indicated by an increase in phase shift values was observed in multiple regions of PSP (red nucleus, putamen, globus pallidus, thalamus) and MSA-p (red nucleus and putamen) patients. Phase values were however unchanged in other brain regions of PD patients.
				Biological gradient	Opposing	Iron	SWI	There were no statistically significant correlations between the SN phase shift values and UPDRS motor scores, or Hoehn-Yahr stages.
Assessment of brain iron and neuronal integrity in patients with Parkinson's disease using novel MRI contrasts	Michaeli et al. 2007[115]	Clinical	High	Strength & consistency	Equivocal	Iron	T2, T2ρ	T2ρ demonstrated a statistically significant difference between the PD and control group, while routine T2 MRI did not.
				Biological gradient	Opposing	Iron	T1ρ, T2ρ	The Spearman correlation between T1ρ and T2ρ in the SN of the PD patients did not correlate with each other.
Usefulness of quantitative susceptibility mapping for the diagnosis of Parkinson disease	Murakami et al. 2015[116]	Clinical	High	Strength & consistency	Supporting	Iron	R2* QSM	Susceptibility mapping values (P=0.001) and R2* (P=0.01) values of the SN were significantly higher in PD patients.
				Specificity	Supporting	Iron	R2* QSM	The QSM and R2* values of the red nucleus, globus pallidus, caudate nucleus, putamen and thalamus were not changed in PD brains compared with controls.

Association of freezing of gait with nigral iron accumulation in patients with Parkinson's disease	Naduthota et al. 2017[117]	Clinical	Moderate	Strength & consistency	Supporting	Iron	R2*	R2* relaxometry scores were increased in the SNc of PD patients, with and without freezing of gait, in comparison with controls.
				Specificity	Opposing	Iron	R2*	Both the SNc and SNr exhibit greater R2* relaxometry values in PD compared with matched controls.
				Biological gradient	Supporting	Iron	R2*	Freezing of gait score had a positive correlation with the R2* value in the caudal SNc (r=0.519, p=0.03).
T1rho and T2rho MRI in the evaluation of Parkinson's disease	Nestrasil et al. 2010[118]	Clinical	High	Strength & consistency	Supporting	Iron	T2ρ	T2ρ relaxation time constants are significantly decreased in PD patients compared with controls (P<0.001).
				Biological gradient	Opposing	Iron	T2ρ	PD patients did not demonstrate a significant correlation between T2ρ signal and UPDRS scores (and subscores), or Hoehn-Yahr stages.
Novel Pattern of Iron Deposition in the Fascicula Nigrale in Patients with Parkinson's Disease: A Pilot Study	Peckham et al. 2016[119]	Clinical	High	Strength & consistency	Supporting	Iron	SWI	There was a significant increase susceptibility of the SN in the PD group (P = 0.012).
MRI supervised and unsupervised classification of Parkinson's disease and multiple system atrophy	Peran et al. 2010[120]	Clinical	Moderate	Strength & consistency	Supporting	Iron	R2*	Patients with PD displayed significantly higher R2* values in the substantia nigra than control subjects.
				Biological gradient	Opposing	Iron	R2*	Regression analysis showed that R2* signals of the left and right SN, are not correlated with UPDRS scores or disease duration.
				Specificity	Supporting	Iron	R2*	A change in R2* value was observed in the SN, but not in other regions including the thalamus, putamen, caudate nucleus, globus pallidus and red nucleus.
High nigral iron deposition in LRRK2 and Parkin mutation carriers using R2* relaxometry	Pyatigorskaya et al. 2015[121]	Clinical	High	Strength & consistency	Equivocal	Iron	R2, R2*	Both symptomatic and asymptomatic carriers of Parkin and LRRK2 mutations reveal a significant increase in R2* value compared with controls, although this is difference is not observed for R2 values.
				Specificity	Supporting	Iron	R2*	No significant differences in R2* values were found in regions outside of the SN.
				Biological gradient	Opposing	Iron	R2*	No significant correlation was found between R2* and UPDRS for symptomatic carriers and healthy controls (P=0.66).
Application of high-field magnetic resonance imaging in Parkinson's disease	Qiao et al. 2017[122]	Clinical	Moderate	Strength & consistency	Supporting	Iron	SWI	Significant reduction in corrected phase values was seen in PD SN compared with controls.
				Specificity	Opposing	Iron	SWI	In addition to the PD SN, differences in corrected phase values were observed in the red nucleus, globus pallidus and putamen.
				Biological gradient	Supporting	Iron	SWI	Corrected phase values of the PD SN, indicative of iron levels, were positively and linearly correlated with FA values, indicative of structural integrity.
Magnetic resonance correlation of iron content with neuromelanin in the substantia nigra of early-stage Parkinson's disease	Reimao et al. 2016[123]	Clinical	High	Strength & consistency	Supporting	Iron	T2*	No difference in T2* signal of SN segments between PD (either early or de novo) and control subjects.
				Biological gradient	Opposing	Iron	T1, T2*	No significant correlation was found between SN neuromelanin volume detected by T1, and iron content detected by T2*.
Clinical MRI for iron detection in Parkinson's disease	Rossi et al. 2013[124]	Clinical	High	Strength & consistency	Equivocal	Iron	R2*, SWI, T2	MRI application in PD patients showed a significant difference in medial SNc R2* values and SWI contrast, but not T2. In the lateral SNc, a significant difference was only observed using R2* in PD patients, but not using T2 and SWI.
				Specificity	Equivocal	Iron	R2*, SWI, T2	SWI and T2-weighted imaging detected a significant increase in iron content of the anterior globus pallidus and posterior globus pallidus respectively. This increase in iron content was not detected using other methods employed in this study.
				Biological gradient	Opposing	Iron	R2*	Pearson correlation analysis shows no correlation between medial SN R2* values and UPDRS-III.

Imaging Brain Iron and Diffusion Patterns. A Follow-up Study of Parkinson's Disease in the Initial Stages	Rossi et al. 2014[125]	Clinical	Moderate	Strength & consistency	Equivocal	Iron	T2, SWI, R2*	No significant difference was observed in SWI contrast or transverse relaxation time R2* in the PD SNc. T2-weighted imaging showed a significant reduction in medial SNc relaxation times of PD patients compared with controls, however no change was detected for the lateral SNc using any of the techniques.
				Specificity	Opposing	Iron	T2, SWI, R2*	Using T2WI and R2*, iron accumulation was observed across multiple structures of the basal ganglia when compared with controls.
				Biological gradient	Opposing	Iron	T2, SWI, R2*	No measurement between SN iron measurement using any of the three MRI techniques and markers of clinical disease severity, including UPDRS.
Magnetic resonance imaging evidence of decreased putamenal iron content in idiopathic Parkinson's disease	Ryvlin et al. 1995[126]	Clinical	Moderate	Strength & consistency	Supporting	Iron	T2	A significant difference in SNc T2 relaxation times was observed between PD patients at different disease stages(<10 years and >10 years) and controls.
				Specificity	Supporting	Iron	T2	The reduction in T2 relaxation times was specific to the SNc, and not other regions within the basal ganglia.
Parkinson's disease related signal change in the nigrosomes 1-5 and the substantia nigra using T2* weighted 7T MRI	Schwarz et al. 2018[127]	Clinical	High	Strength & consistency	Supporting	Iron	T2*	Average T2*w signal values were significantly reduced across all nigrosomes and in the iron-rich SN of PD patients.
				Biological gradient	Supporting	Iron	T2*	Significant correlations of nigrosomal T2* signals with UPDRS were found, where nigrosome 1 (R^2=0.426), nigrosomes 2-5 (R^2=0.335), and iron-rich SN (R^2=0.214).
Iron quantification in Parkinson's disease using an age-based threshold on susceptibility maps: The advantage of local versus entire structure iron content measurements	Sethi et al. 2019[128]	Clinical	Moderate	Strength & consistency	Supporting	Iron	QSM	Idiopathic PD group showed larger susceptibility in the RII SN compared with healthy controls in both the right and left side, as well as for whole-region susceptibility.
				Specificity	Equivocal	Iron	QSM	There was a significant increase in mean RII susceptibilities of the red nucleus in both the left and right sides of PD patients compared with controls. Whole-region red nucleus mean susceptibility did not show a significant difference.
				Biological gradient	Opposing	Iron	QSM	Weak, insignificant correlations were demonstrated between mean RII susceptibility values and disease state scores.
Evaluation of iron deposition in brain basal ganglia of patients with Parkinson's disease using quantitative susceptibility mapping	Shahmaei et al. 2019[129]	Clinical	Moderate	Strength & consistency	Supporting	Iron	QSM	QSM analysis demonstrated a significant increase in iron deposition in PD SN compared with matched controls.
				Specificity	Opposing	Iron	QSM	Susceptibility values are significantly changed in the SN, red nucleus, globus pallidus and thalamic nuclei between PD and controls.
				Biological gradient	Supporting	Iron	QSM	A significant correlation between mean SN susceptibility values and Hoehn-Yahr stages was found (r=0.751, P<0.001).
Non-Motor Symptom Burdens Are Not Associated with Iron Accumulation in Early Parkinson's Disease: a Quantitative Susceptibility Mapping Study	Shin et al. 2018[130]	Clinical	Moderate	Strength & consistency	Opposing	Iron	QSM	Quantitative susceptibility mapping did not detect any changes between PD SN versus controls.
				Biological gradient	Opposing	Iron	QSM	The severity of non-motor symptoms in PD patients, determined by K-NMSS total score, was not correlated to SN susceptibility. Disease duration, Hoehn-Yahr stage, UPDRS-III score were not correlated with SN QSM values either.
Quantitative susceptibility mapping differentiates between parkinsonian disorders	Sjostrom et al. 2017[131]	Clinical	Moderate	Strength & consistency	Supporting	Iron	QSM	PD SN susceptibility is significantly increased in comparison to the control group (P=0.030).
				Specificity	Equivocal	Iron	QSM	Mean susceptibilities are not significantly changed in the globus pallidus, putamen and red nucleus of the PD group. Evidence of increased susceptibility was however observed in MSA and PSP brains.
				Biological gradient	Opposing	Iron	QSM	Regression analysis showed no association between susceptibility levels in the SN and higher Hoehn-Yahr stages (P=0.454).
T2*-weighted MRI values correlate with motor and cognitive dysfunction in Parkinson's disease	Tambasco et al. 2019[132]	Clinical	High	Strength & consistency	Opposing	Iron	T2*	No significant difference between T2*-weighted values in PD SN and controls was found.
				Biological gradient	Supporting	Iron	T2*	Iron content of the SN correlated positively with disease duration and UPDRS-III off scores, whilst it was inverse correlated with Montreal Cognitive Assessment, Spatial Span, Graded Naming Test and Wechsler Adult Intelligence Scale-IV.

Voxel-based quantitative susceptibility mapping in Parkinson's disease with mild cognitive impairment	Uchida et al. 2019[133]	Clinical	High	Strength & consistency	Supporting	Iron	QSM	The QSM value in the SN was significantly increased in PD patients with normal cognition and mild cognitive impairment when compared with healthy controls.
				Specificity	Opposing	Iron	QSM	Mapping susceptibility was significantly elevated in SN, globus pallidus, putamen, caudate nucleus, entorhinal cortex, parahippocampal gyrus, orbitofrontal cortex, amygdala and precuneus of PD patients compared with controls.
				Biological gradient	Opposing	Iron	QSM	No significant correlations were observed between SN PD susceptibility values and UPDRS-III.
Is R2* a new MRI biomarker for the progression of Parkinson's disease? A longitudinal follow-up	Ulla et al. 2013[134]	Clinical	High	Strength & consistency	Supporting	Iron	R2*	A significant increase in transverse relaxation rate R2* was seen in the PD SNc, compared with controls. Longitudinal analysis over three years showed a 10.2% (p=0.001) increase in the PD SNc R2* relaxation rate.
				Specificity	Opposing	Iron	R2*	R2* values were increased in the PD SNc, and SNr compared with controls at baseline. A three year follow-up study showed a significant increase in the SNc and putamen R2* signal in PD patients, although this evolution was not observed in controls.
				Biological gradient	Supporting	Iron	R2*	A positive correlation between the ΔR2* signal in the SNc over three years and ΔUPDRS scores was found in PD patients (R=0.586, p=0.028).
T1 and T2 in the brain of healthy subjects, patients with Parkinson disease, and patients with multiple system atrophy: Relation to iron content	Vymazal et al. 1999[135]	Clinical	Moderate	Strength & consistency	Opposing	Iron	T2	T2 relaxation times are not altered between PD SNc and control subjects.
				Analogy	Supporting	Iron	T1, T2	There was a concomitant reduction in T1 and T2 signals in MSA globus pallidus indicating regional atrophy and iron accumulation respectively.
MRI assessment of basal ganglia iron deposition in Parkinson's disease	Wallis et al. 2008[136]	Clinical	Moderate	Strength & consistency	Supporting	Iron	R2'	There was a significant increase in mean R2' relaxation rate in the SN for patients with PD.
				Specificity	Opposing	Iron	R2'	Significant group differences were shown for the substantia nigra, and putamen with higher R2' in the PD group.
				Biological gradient	Supporting	Iron	R2'	The UPDRS motor symptom severity score for the PD patients's most affected side was positively correlated with the contralateral SN R2' signal (r=0.292, P=0.035).
Quantitative assessment of iron deposition in the midbrain using 3D-enhanced T2 star weighted angiography (ESWAN): A preliminary cross-sectional study of 20 Parkinson's disease patients	Wang et al. 2013[137]	Clinical	High	Strength & consistency	Supporting	Iron	T2* (ESWAN)	Mean phase values measured using T2*-weighted magnetic resonance demonstrated a significant reduction in patients with mild PD (H-Y stage <2.5) compared with controls. A further significant reduction is seen in PD patients with moderate to severe impairment (H-Y stage 3.0) compared with mild PD.
				Specificity	Opposing	Iron	T2* (ESWAN)	Reduction in mean phase value was recorded in the PD SNr compared with controls.
				Biological gradient	Supporting	Iron	T2* (ESWAN)	SNc region mean phase values were shown to be negatively correlated with Hoehn and Yahr staging in the minimal impairment (H-Y stage <2.5) and moderate to severe impairment (H-Y stage >3) PD groups.
Freezing of gait in early Parkinson's disease: Nigral iron content estimated from magnetic resonance imaging	Wieler et al. 2016[138]	Clinical	High	Specificity	Opposing	Iron	R2*	Longitudinal R2* values were significantly different in the SNr and SNc compared with baseline.
				Biological gradient	Supporting	Iron	R2*	There was a significant correlation between the change in the UPDRS III score from baseline to 36 months, and the measured R2* values (36 months - baseline) in the lateral SNc of PD patients.
Longitudinal midbrain changes in early Parkinson's disease: iron content estimated from R2*/MRI	Wieler et al. 2015[139]	Clinical	High	Strength & consistency	Supporting	Iron	R2*	A difference in R2* values was observed in the lateral SNc between PD and control subjects at baseline (p = 0.035), though no significant difference was observed after three years of follow-up.
				Specificity	Supporting	Iron	R2*	A difference in R2* values at baseline was seen between PD and controls in the lateral SNc which is more vulnerable to degeneration compared with the media SNc or the SNr.

				Biological gradient	Equivocal	Iron	R2*	In PD patients, there were significant correlations between the measured R2* change in the lateral SNc and the change in score in UPDRS III (P=0.008) from baseline to 36 months. This correlation was also found with the PDQ-39 mobility subscore (P=0.03), but not TUG time change, 14m speed change and Berg total change.
Assessment of cerebral iron content in patients with Parkinson's disease by the susceptibility-weighted MRI	Wu et al. 2014[140]	Clinical	Moderate	Strength & consistency	Supporting	Iron	SWI	Early stage PD, and intermediate and advanced PD patients showed a significant difference in SN corrected values in comparison with controls.
				Specificity	Opposing	Iron	SWI	Early, intermediate and advanced stage PD patients showed a significant difference in corrected phase values across basal nuclei (SN, caudate nucleus, putamen and globus pallidus). Another significant difference was also seen in the red nucleus of intermediate and advanced PD patients.
				Biological gradient	Supporting	Iron	SWI	Correlation analysis demonstrated that phase values significantly and inversely correlated with the Hoehn-Yahr scale in the SN (R=-0.620, P=0.032).
Different iron deposition patterns in early- and middle-late-onset Parkinson's disease	Xuan et al. 2017[141]	Clinical	High	Strength & consistency	Supporting	Iron	QSM	Regional susceptibility values demonstrated a significant increase in the SNc of early-onset PD patients and mid-late onset PD patients when compared with age-matched controls.
				Specificity	Opposing	Iron	QSM	Early-onset PD showed increased susceptibility values in the PD SNr, and mid-late onset PD patients demonstrated an increase in putaminal iron content.
				Biological gradient	Equivocal	Iron	QSM	Mid-late onset PD patients showed positively correlations between Hoehn-Yahr stages (r=0.474, P=0.007), UPDRS II scores and susceptibility values in the SNc. No significant correlations were observed between susceptibility values and clinical features in early-onset PD patients.
Characterizing iron deposition in Parkinson's disease using susceptibility-weighted imaging: An in vivo MR study	Zhang et al. 2010[142]	Clinical	High	Strength & consistency	Supporting	Iron	SWI	The SN, located in the hemisphere contralateral to the most affected body side of symptoms assessed with UPDRS, showed a significant difference in phase shift values between PD patients and controls.
				Specificity	Supporting	Iron	SWI	Comparison of the phase shift values between the most affected brain side in PD and healthy controls that a significant increase is restricted to the SN, and not other regions including the red nucleus, caudate nucleus, putamen, globus pallidus, thalamus and frontal white matter.
				Biological gradient	Supporting	Iron	SWI	Correlation analysis demonstrated that there was a positive correlation between the phase shift values between UPDRS motor scores and the phase shift values in the SN of the most affected (r=0.412, P=0.008) and least affected brain sides (r=0.361, P=0.022).
Determination of brain iron content in patients with Parkinson's disease using magnetic susceptibility imaging	Zhang et al. 2009[143]	Clinical	Moderate	Strength & consistency	Supporting	Iron	SWI	Both subgroups of PD patients, characterised with or without cognitive dysfunction, showed a significant decrease in phase radians compared with controls.
				Specificity	Opposing	Iron	SWI	There was a significant difference in the phase radians of SNc, caudate nucleus and red nucleus between PD patients and controls.
				Biological gradient	Supporting	Iron	SWI	A positive correlation between UPDRS motor scores and SN phase radians in the most affected side in PD patients was observed (P<0.05).
Quantitative susceptibility mapping in Parkinson's disease	Langkammer et al. 2016[144]	Clinical	High	Strength & consistency	Supporting	Iron	QSM R2*	Regional mean susceptibilities and R2* values show a significant increase in PD SN compared with controls.
				Specificity	Equivocal	Iron	QSM R2*	Mean susceptibility values showed a significant increase in the SN, nucleus ruber and thalamus compared with controls. However, transverse relaxation rate R2* is only increased in the SN, but not other regions.
				Biological gradient	Supporting	Iron	QSM R2*	A significant positive correlation was seen between SN R2* and magnetic susuceptibilites with Hoehn-Yahr staging, UPDRS-I, UPDRS-II and UPDRS-total.
3D texture analyses within the substantia nigra of Parkinson's disease patients on quantitative susceptibility maps and R2* maps	Li et al. 2019[145]l	Clinical	Moderate	Strength & consistency	Equivocal	Iron	QSM R2*	3D texture analysis of PD SN showed a significant increase in mean susceptibility values, but not R2* values.
				Biological gradient	Opposing	Iron	QSM R2*	There was no correlation between mean QSM and R2* values in the SN and UPDRS-III scores.

Volume of Interest Analysis of Spatially Normalized PRESTO Imaging to Differentiate between Parkinson Disease and Atypical Parkinsonian Syndrome	Sakurai et al. 2016[146]	Clinical	High	Strength & consistency	Opposing	Iron	PRESTO imaging	No change in signal intensity ratio was observed between PD and controls.
				Specificity	Supporting	Iron	PRESTO imaging	Iron accumulation was not detected in the SN of MSA-P patients compared with healthy controls.
Transcranial ultrasound shows nigral hypoechogenicity in restless legs syndrome	Schmidauer et al. 2005[147]	Clinical	High	Strength & consistency	Supporting	Iron	TCS	Total area of hypechogenicity observed in the PD SN is significantly increased compared with controls.
Comprehensive MRI quantification of the substantia nigra pars compacta in Parkinson's disease	Takahashi et al. 2018[148]	Clinical	Moderate	Strength & consistency	Supporting	Iron	QSM	The QSM value was significantly higher in the PD group than in the healthy control group.
				Biological gradient	Supporting	Iron	QSM	Correlative analysis showed a relationship between iron accumulation measured by QSM and neuromelanin volume in the PD SNc (r=0.38).
Longitudinal evaluation of iron concentration and atrophy in the dentate nuclei in friedreich ataxia	Ward et al. 2019[149]	Clinical	Moderate	Analogy	Supporting	Iron	QSM	Friedrich's ataxia patients show a concomitant 31% elevation of iron content and a 26% reduction in the volume of dentate nuclei. A similar pattern was seen after a two-year follow-up.
Quantitative assessment of brain iron by R2* relaxometry in patients with cervical dystonia	Aschermann et al. 2015[150]	Clinical	Moderate	Analogy	Opposing	Iron	R2*	Cervical dystonia - R2* relaxation rates demonstrated an 8% increase in globus pallidus iron content (P=0.002) although this was not associated with a change in regional volume.
Iron accumulation and dysregulation in the putamen in fragile X-associated tremor/ataxia syndrome	Ariza et al. 2017[151]	Post-mortem	Moderate	Analogy	Supporting	Iron	Perl's staining	There was a marginal increase in neuronal iron concentrations (+12%) in FXTAS patients compared with age-matched controls
Significance of the hot-cross bun sign on T2*-weighted MRI for the diagnosis of multiple system atrophy	Deguchi et al. 2015[152]	Clinical	High	Analogy	Supporting	Iron	T2	Reduced T2 relaxation times were identified in the degenerating putamen in MSA brains, compared with controls.
Quantitative assessment of subcortical atrophy and iron content in progressive supranuclear palsy and parkinsonian variant of multiple system atrophy	Lee et al. 2013[153]	Clinical	High	Analogy	Supporting	Iron	R2*	In MSA-p patients, there is a strong inverse correlation between R2* relaxation value and volume of the putamen (r=-0.777, P<0.001) and globus pallidus (r=-0.409, P=0.025). A similar association is seen between R2* values and volume of the globus pallidus in PSP patients (r=0.400, P=0.043), but not in the putamen.
Progression of subcortical atrophy and iron deposition in multiple system atrophy: a comparison between clinical subtypes	Lee et al. 2015[154]	Clinical	High	Analogy	Supporting	Iron	R2*	A longitudinal study on MSA-p patients demonstrated a concomitant 16% increase in putaminal iron content and a 9% reduction in putaminal volume after 18 months.
Expression and activity of antioxidants in the brain in progressive supranuclear palsy	Cantuti-Castelvetri et al. 2002[155]	Post-mortem	Moderate	Analogy	Supporting	Copper	Protein assays	Marked increases in SOD1 activity and glutathione levels were reported in the degenerating putamen of PSP patients.

Supplementary Table 1. Outcome of the Bradford Hill analysis conducted on each article included from our systematic search. *Abbreviations: AAS, atomic absorption spectroscopy; GF-AAS, graphite furnace-atomic absorption spectroscopy; WB, western blot; ICP-MS, inductively coupled plasma-mass spectrometry; IHC, immunohistochemistry; SXFM, synchrotron X-ray fluorescence microscopy; ROS, reactive oxygen species; HPLC, high performance liquid chromatography; TIBC, total iron binding capacity; ELISA, enzyme-linked immunosorbent assay; TCS, transcranial sonography; PIXE, particle-induced X-ray emission; MRI, magnetic resonance imaging; UPDRS, Unified Parkinson's Disease Rating Scale; RIA, radioimmunoassay; CP, ceruloplasmin; SOD1, superoxide dismutase 1; IEF, isoelectric focussing; PCR, polymerase chain reaction; 2D-PAGE, two-dimensional polyacrylamide gel electrophoresis; PCR-RFLP, PCR-restriction fragment length polymorphism; DHPLC, denaturing high performance liquid chromatography; qRT-PCR, quantitative reverse transcription-PCR; HPLC-UV, HPLC-ultraviolet; QSM, quantitative susceptibility mapping; FDRI, field dependent relaxation rate increase; SWI, susceptibility weighted imaging; ESWAN, enhanced susceptibility-weighted angiography; PRESTO, principles of echo shifting with a train of observations; PD, Parkinson's disease; SN, substantia nigra; SNc, substantia nigra pars compacta; SNr, substantia nigra pars reticulata; DLB, dementia with Lewy bodies; LC, locus coeruleus; MSA, multiple system atrophy; AD, Alzheimer's disease; HD, Huntington's disease; PSP, progressive supranuclear palsy; LBD, Lewy body disease; ILBD, incidental Lewy body disease; TH, tyrosine hydroxylase; CTR1, copper transport protein 1; IRP, iron regulatory protein; IRE, iron responsive element; mRNA, messenger ribonucleic acid; DMT1, divalent metal transporter 1; GFAP, glial fibrillary acidic protein; HO-1, hemeoxygenase-1; VNTR, variable number tandem repeat; SNP, single nucleotide polymorphism; HFE, haemochromatosis; DNA, deoxyribonucleic acid; TF, transferrin; transferrin receptor, transferrin receptor; MMSE, mini-mental state examination; H-Y, Hoehn-Yahr; PDQ-39, The Parkinson's Disease Questionnaire; CSF, cerebrospinal fluid; LRRK2, leucine-rich repeat kinase 2; RII, high-iron region; K-NMSS, Korean–Non-motor Symptoms Scale for Parkinson's Disease; TUG, Timed Up and Go Test; MSA-P, MSA-parkinsonism; FXTAS, Fragile X-associated tremor/ataxia syndrome.*

Study quality												
Criterion	High and moderate quality				High quality				Moderate quality			
	Supporting	Opposing	Equivocal	Conclusion	Supporting	Opposing	Equivocal	Conclusion	Supporting	Opposing	Equivocal	Conclusion
Strength & consistency	92	30	10	Supporting	45	14	6	Supporting	47	16	4	Supporting
Specificity	32	29	12	Equivocal	19	13	5	Supporting	13	16	7	Equivocal
Plausibility	6	2	0	Supporting	3	0	0	Supporting	3	2	0	Equivocal
Biological gradient	35	27	9	Supporting	19	13	7	Supporting	16	14	2	Equivocal
Analogy	11	1	1	Supporting	4	0	0	Supporting	7	1	1	Supporting
Temporality	9	2	0	Supporting	4	0	0	Supporting	5	2	0	Supporting
Experiment	2	1	0	Equivocal	2	1	0	Equivocal	0	0	0	No data
Coherence	188	91	32	Supporting	97	40	18	Supporting	91	51	14	Supporting

Investigation type												
Criterion	Clinical investigations				Post-mortem investigations				Protein/genetic studies excluded			
	Supporting	Opposing	Equivocal	Conclusion	Supporting	Opposing	Equivocal	Conclusion	Supporting	Opposing	Equivocal	Conclusion
Strength & consistency	66	26	8	Supporting	26	4	2	Supporting	78	10	8	Supporting
Specificity	20	21	9	Equivocal	12	8	3	Supporting	28	25	12	Equivocal
Plausibility	2	0	0	Supporting	4	2	0	Supporting	6	1	0	Supporting
Biological gradient	32	24	9	Supporting	3	3	0	Equivocal	33	24	9	Supporting
Analogy	6	1	0	Supporting	5	0	1	Supporting	9	1	1	Supporting
Temporality	7	0	0	Supporting	2	2	0	Equivocal	2	1	0	Equivocal
Experiment	2	1	0	Equivocal	0	0	0	No data	2	1	0	Equivocal
Coherence	136	72	26	Supporting	52	19	6	Supporting	158	63	30	Supporting

Supplementary Table 2. Conclusions drawn from application of the Bradford Hill model to studies investigating the etiological role of iron in Parkinson's disease. Studies were stratified according to quality using the Genoud Scale and NIH Quality Assessment Tools, and investigation type, noting that data reported here includes both high and moderate quality studies.

Criterion	High and moderate quality				High quality				Moderate quality			
	Supporting	Opposing	Equivocal	Conclusion	Supporting	Opposing	Equivocal	Conclusion	Supporting	Opposing	Equivocal	Conclusion
**Study quality**												
Strength & consistency	9	13	1	Opposing	5	4	1	Equivocal	4	9	0	Opposing
Specificity	5	1	0	Supporting	2	1	0	Equivocal	3	0	0	Supporting
Plausibility	3	1	0	Supporting	2	0	0	Supporting	1	1	0	Equivocal
Biological gradient	3	1	0	Supporting	2	1	0	Equivocal	1	0	0	Equivocal
Analogy	3	0	0	Supporting	1	0	0	Equivocal	2	0	0	Supporting
Temporality	3	1	0	Supporting	3	0	0	Supporting	0	1	0	Equivocal
Experiment	0	0	0	No data	0	0	0	No data	0	0	0	No data
Coherence	26	17	1	Supporting	15	6	1	Supporting	11	11	0	Equivocal

Criterion	Clinical investigations				Post-mortem investigations				Protein/genetic studies excluded			
	Supporting	Opposing	Equivocal	Conclusion	Supporting	Opposing	Equivocal	Conclusion	Supporting	Opposing	Equivocal	Conclusion
**Investigation type**												
Strength & consistency	2	5	0	Opposing	7	8	1	Equivocal	5	3	0	Supporting
Specificity	0	0	0	Equivocal	5	1	0	Supporting	5	1	0	Supporting
Plausibility	0	0	0	Equivocal	3	1	0	Supporting	2	0	0	Supporting
Biological gradient	0	1	0	Equivocal	3	0	0	Supporting	2	0	0	Supporting
Analogy	0	0	0	Equivocal	3	0	0	Supporting	2	0	0	Supporting
Temporality	1	0	0	Equivocal	2	1	0	Equivocal	1	0	0	Equivocal
Experiment	0	0	0	Equivocal	0	0	0	No data	0	0	0	No data
Coherence	3	6	0	Opposing	23	11	1	Supporting	17	4	0	Supporting

Supplementary Table 3. Conclusions drawn from application of the Bradford Hill model to studies investigating the etiological role of copper in Parkinson's disease. Studies were stratified according to quality using the Genoud Scale and NIH Quality Assessment Tools, and investigation type, noting that data reported here includes both high and moderate quality studies.

Criteria	Outcome	AAS		ICP-MS	
		Iron	Copper	Iron	Copper
**Strength & consistency**	Supporting	4	1	5	4
	Opposing	1	3	1	0
	Equivocal	1	0	0	0
**Specificity**	Supporting	2	1	3	3
	Opposing	2	1	0	0
	Equivocal	2	0	0	0
**Plausibility**	Supporting	1	1	0	0
	Opposing	0	0	1	0
	Equivocal	0	0	0	0
**Biological gradient**	Supporting	0	0	0	0
	Opposing	0	0	0	0
	Equivocal	0	0	0	0
**Analogy**	Supporting	3	1	0	0
	Opposing	0	0	0	0
	Equivocal	0	0	0	0
**Temporality**	Supporting	0	0	0	0
	Opposing	1	0	0	0
	Equivocal	0	0	0	0
**Experiment**	Supporting	0	0	0	0
	Opposing	0	0	0	0
	Equivocal	0	0	0	0
**Coherence**	Supporting	10	4	8	7
	Opposing	4	4	2	0
	Equivocal	3	0	0	0

Supplementary Table 4. Comparison of Bradford Hill criteria outcomes for studies using atomic absorption spectroscopy or inductively coupled plasma–mass spectrometry.

Criteria	Outcome	Ferritin	Ferroportin	Prohibitin	Transferrin	Lactoferrin	DMT1	HO-1	SOD1	HFE	IRP-2	GSH	CP
**Strength & consistency**	Supporting	4	0	1	3	1	2	2	2	1	0	1	2
	Opposing	3	0	0	5	1	1	2	6	7	3	1	3
	Equivocal	3	1	1	0	0	0	0	1	0	0	1	0
**Specificity**	Supporting	1	0	0	1	1	0	1	0	0	0	0	0
	Opposing	3	0	0	1	0	0	0	0	0	0	0	1
	Equivocal	0	1	1	0	0	0	0	0	0	0	0	0
**Plausibility**	Supporting	0	0	0	0	0	0	0	2	0	0	0	1
	Opposing	2	0	0	0	0	0	0	0	0	0	0	0
	Equivocal	0	0	0	0	0	0	0	0	0	0	0	0
**Biological gradient**	Supporting	0	0	0	1	1	0	0	1	0	0	1	0
	Opposing	0	0	0	2	0	0	0	0	2	0	0	1
	Equivocal	0	0	0	0	0	0	0	0	0	0	0	0
**Analogy**	Supporting	2	0	0	0	0	0	0	1	1	0	1	2
	Opposing	0	0	0	0	0	0	0	0	0	0	0	0
	Equivocal	0	0	0	0	0	0	0	0	0	0	0	0
**Temporality**	Supporting	0	0	0	2	0	1	1	1	1	0	0	1
	Opposing	0	0	0	0	0	0	0	0	0	0	2	0
	Equivocal	0	0	0	0	0	0	0	0	0	0	0	0
**Experiment**	Supporting	0	0	0	0	0	0	0	0	0	0	0	1
	Opposing	0	0	0	0	0	0	0	0	0	0	0	0
	Equivocal	0	0	0	0	0	0	0	0	0	0	0	0
**Coherence**	Supporting	8	0	1	7	3	3	5	7	3	0	3	7
	Opposing	8	0	0	8	1	1	1	6	9	3	3	5
	Equivocal	4	2	2	0	0	0	0	1	0	0	1	0

Supplementary Table 5. Bradford Hill analysis performed on iron and copper proteins/genes in Parkinson's disease. Red indicates criteria with a supporting or opposing outcome.

Database	Fields	Search Strategy	Filter	Generated Articles
**Pubmed**	All text	(Parkinson* OR "Parkinson*"[mh]) AND (copper OR iron OR Cu OR Fe OR "copper protein?" OR cupro* OR "iron protein?" OR ferro*)	Human	2114
**Ovid EMBASE**	All text	(Parkinson*) and (copper or iron or Cu or Fe or "copper protein?" or cupro* or "iron protein?" OR ferro*)	Human	4154
**CENTRAL**	All text	(Parkinson*) AND (Copper OR iron OR Cu OR Fe OR copper protein? OR cupro* OR iron protein? OR ferro*)	-	176
**Scopus**	Title, abstract, keyword	(Parkinson*) AND (Copper OR Iron OR Cu OR Fe OR "copper protein?" OR cupro* OR "iron protein?" OR ferro*) AND (Human)	Article	1993
			**Total**	8437

Supplementary Table 6. Number of generated articles using our search strategy for four unique databases. The database search was conducted on September 19th, 2019.

Stage	Filter	Duplicates Removed
Stage 1	Author, Year, Title, Journal	1297
Stage 2	Author, Year, Title, Pages	398
Stage 3	Title, Journal, Pages	258
Stage 4	Year, Title, Pages	75
Stage 5	Title, Pages	0
Stage 6	Author, Year, Journal, Pages	0
Stage 7	Author, Year, Title	769
Stage 8	Author, Year, Journal	196
Stage 9	Author, Year	167
Stage 10	Title	276
Stage 11	No filter set; articles were sorted by Title and duplicates were removed	85
Stage 12	Remaining duplicates removed during abstract screening	26
	**Total after duplicate removal**	4891

Supplementary Table 7. Duplicates removed at each stage in a comprehensive 12-step process using EndNote 9. A unique filter was set at each stage to identify previously undetected duplicate articles.

Criteria	Classification	Description
Not English	Excluded	Article is not written in English
Publication type	Excluded	Book chapters, reviews, systematic reviews, meta analyses, methods papers, conference abstracts, conference proceedings, editorial letters and responses
Animal & plant studies	Excluded	Non-interventional study of animal models and all plant studies
In vitro & in silico studies	Excluded	Article uses in vitro or in silico models
Not PD, PD syndromes or diseases analogous to PD	Excluded	Research article that does not investigate Parkinson's disease, Parkinsonian syndromes, or diseases analogous to PD; co-morbidities are to be excluded
Not iron or copper	Excluded	Article topic involved PD, but outside the scope of iron and copper metal changes
Small sample size	Excluded	Total sample size is less than four, or is a single case study
Repeated data	Excluded	Data sets that exist in multiple articles
Non-degenerating regions	Excluded	Article investigates changes in copper or iron metal, protein and/or gene levels in a non-degenerating region in PD brains
Not copper/iron targeting therapy	Excluded	Human interventional studies that do not use copper or iron (metal or protein) modulating treatments
Metal exposure	Excluded	Exposure to heavy, essential and trace metals via environmental factors (e.g. work, diet, etc.)
In vivo imaging -- no metals	Excluded	In vivo imaging in degenerating PD regions that does not include metals
Protein/gene irrelevant	Excluded	Article investigated a protein/gene in degenerating PD tissue, but they are not involved in copper/iron handling and metabolism
In vivo human brain imaging	Included	Quantitative or semi-quantitative in vivo imaging of iron/copper metal or protein levels in degenerating Parkinson's disease brain regions
Post-mortem metal levels	Included	Quantitative or semi-quantative analysis of iron/copper in in post-mortem degenerating Parkinson's disease brain regions
Protein/genetic changes	Included	Iron and copper protein expression and activity, as well as relevant transcriptome changes (i.e. mRNA expression, gene mutations, polymorphisms) in degenerating PD brain regions
Analogous to PD	Included	Diseases reflecting changes in iron and copper metal, protein and/or gene levels reported in Parkinson's disease (CNS or PNS degeneration). Interventional studies that assess the therapeutic benefits of the drug (i.e. not pharmacokinetic studies) fell in this category.

Supplementary Table 8. Predefined inclusion and exclusion criteria for the Bradford Hill systematic analysis. Abbreviations: PD, Parkinson’s disease; CNS, central nervous system; PNS, peripheral nervous system.

Category	Definition
Iron proteins	Tyrosine hydroxylase, ceruloplasmin (ferroxidase activity), divalent metal transport 1, ferritin, ferroportin, lactoferrin, lactoferrin receptor, transferrin, transferrin receptor, heme oxygenase-1, myeloperoxidase, hemoglobin, 20S Proteasome, cytochrome c oxidase, complexes II & III, prohibitin, catalase, hepcidin, iron regulatory protein, human homeostatic iron regulator protein
Copper proteins	Copper chaperone for superoxide dismutase, superoxide dismutase 1/3, catalase, glutathione peroxidase, monoamine oxidase, dopamine beta-hydroxylase, ceruloplasmin, NADH dehydrogenase, cytochrome c oxidase, ubiquitin, 20S, proteasome, alpha-synuclein, metal regulatory transcription factor 1, copper transport protein 1, metallothionein ½, ATP7A/B, glutathione, ATOX1, Sp-1

Supplementary Table 9. List of iron- and copper-related proteins considered in the scope of this systematic review.

Selection (6 points)	
**1. Is the case definition adequate?**	
a. Both clinically and pathologically defined	2
b. Either clinically or pathologically defined	1
c. No description	0
**2. Representation of population**	
a. Cases from multiple tissue banks or live cohorts	2
b. Tissue bank or live cohort stated	1
c. Potential for selection biases or not stated	0
**3. Selection of controls**	
a. Controls selected from same tissue banks or live population	1
b. Controls from different source or no description	0
**4. Definition of controls**	
a. No history of neurological disease or confounding condition	1
b. Diagnosed conditions provided but effect on variable of interest unknown	0
c. No description	0
**Comparability (5 points)**	
**1. Comparability of cases and controls on the basis of the design or analysis**	
a. Study controls for the most significant potential moderating variables x and y (e.g. age, sex, genetic background, PMI)	2
b. Study controls for only one (most important) factor	1
c. Not controlled or not stated	0
**2. Quality of tissue**	
a. Post-mortem delay is <24hrs for post-mortem tissues; or biofluids were stored at -20°C <1hr after collection (1 point)	1
b. >24hrs for post-mortem or >1hr for biofluids or not defined	0
**3. Method detail**	
a. An appropriate method applied and described	2
b. Methods and/or description limited	1
c. Inappropriate method and/or missing crucial information	0
**Analysis (5 points)**	
**1. Ascertainment of exposure**	
a. Blinded to case/control status	1
b. Unblinded	0
c. No description	0
**2. Appropriate and detailed statistical analysis**	
a. Detailed and appropriate analysis	2
b. Statistical tested stated	1
c. No description	0
**3. Elimination of outliers**	
a. Appropriate outlier analyses were conducted, and similar proportion of participants were excluded from all groups	2
b. Appropriate outlier analyses were conducted, and different proportion of participants were excluded from all groups	1
c. No outlier analyses conducted	0
**Maximum score – selection, comparability & analysis**	**16**

Supplementary Table 10. Revised scoring rubric for the Quality Assessment Scale for Biochemical Analysis of Human Samples (Genoud scale). We amended the ‘elimination of outliers’ category to account for the differential exclusion of outliers from experimental groups, allowing for a maximum of 2 allottable scores for this category, and 16 for the entire scale.

References

1. Ayton S, Lei P, Duce JA, et al. Ceruloplasmin dysfunction and therapeutic potential for Parkinson disease. *Ann Neurol*. 2013;73(4):554-559. doi:10.1002/ana.23817

2. Faucheux BA, Martin M-E, Beaumont C, Hauw J-J, Agid Y, Hirsch EC. Neuromelanin associated redox-active iron is increased in the substantia nigra of patients with Parkinson's disease. *J Neurochem*. 2003;86(5):1142-1148. doi:10.1046/j.1471-4159.2003.01923.x

3. Hirsch EC, Brandel J -P, Galle P, Javoy-Agid F, Agid Y. Iron and Aluminum Increase in the Substantia Nigra of Patients with Parkinson's Disease: An X-Ray Microanalysis. *J Neurochem*. 1991;56(2):446-451. doi:10.1111/j.1471-4159.1991.tb08170.x

4. Howitt J, Gysbers AM, Ayton S, et al. Increased Ndfip1 in the substantia nigra of parkinsonian brains is associated with elevated iron levels. *PLoS One*. 2014;9(1). doi:10.1371/journal.pone.0087119

5. Jellinger K, Paulus W, Grundke-Iqbal I, Riederer P, Youdim MBH. Brain iron and ferritin in Parkinson's and Alzheimer's diseases. *J Neural Transm - Park Dis Dement Sect*. 1990;2(4):327-340. doi:10.1007/BF02252926

6. Jellinger K, Kienzl E, Rumpelmair G, et al. Iron-Melanin Complex in Substantia Nigra of Parkinsonian Brains: An X-Ray Microanalysis. *J Neurochem*. 1992;59(3):1168-1171. doi:10.1111/j.1471-4159.1992.tb08362.x

7. Szczerbowska-Boruchowska M, Krygowska-Wajs A, Adamek D. Elemental micro-imaging and quantification of human substantia nigra using synchrotron radiation based x-ray fluorescence - In relation to Parkinsons disease. *J Phys Condens Matter*. 2012;24(24):244104-244114. doi:10.1088/0953-8984/24/24/244104

8. Uitti RJ, Rajput AH, Rozdilsky B, Bickis M, Wollin T, Yuen WK. Regional Metal Concentrations in Parkinson's Disease, Other Chronic Neurological Diseases, and Control Brains. *Can J Neurol Sci*. 1989;16(3):310-314. doi:10.1017/S0317167100029140

9. Visanji NP, Collingwood JF, Finnegan ME, Tandon A, House E, Hazrati L-N. Iron deficiency in parkinsonism: Region-specific iron dysregulation in parkinson's disease and multiple system atrophy. *J Parkinsons Dis*. 2013;3(4):523-537. doi:10.3233/JPD-130197

10. Wypijewska A, Galazka-Friedman J, Bauminger ER, et al. Iron and reactive oxygen species activity in parkinsonian substantia nigra. *Park Relat Disord*. 2010;16(5):329-333. doi:10.1016/j.parkreldis.2010.02.007

11. Riederer P, Sofic E, Rausch W -D, et al. Transition Metals, Ferritin, Glutathione, and Ascorbic Acid in Parkinsonian Brains. *J Neurochem*. 1989;52(2):515-520. doi:10.1111/j.1471-4159.1989.tb09150.x

12. Oakley AE, Collingwood JF, Dobson J, et al. Individual dopaminergic neurons show raised iron levels in Parkinson disease. *Neurology*. 2007;68(21):1820 LP - 1825. http://n.neurology.org/content/68/21/1820.abstract.

13. Mann VM, Cooper JM, Daniel SE, et al. Complex I, iron, and ferritin in Parkinson's disease substantia nigra. *Ann Neurol*. 1994;36(6):876-881. doi:10.1002/ana.410360612

14. Loeffler DA, Connor JR, Juneau PL, et al. Transferrin and iron in normal, Alzheimer's disease, and Parkinson's disease brain regions. *J Neurochem*. 1995;65(2):710-724. doi:10.1046/j.1471-4159.1995.65020710.x

15. Loeffler DA, LeWitt PA, Juneau PL, et al. Increased regional brain concentrations of ceruloplasmin in neurodegenerative disorders. *Brain Res*. 1996;738(2):265-274. doi:10.1016/S0006-8993(96)00782-2

16. Genoud S, Roberts BR, Gunn AP, et al. Subcellular compartmentalisation of copper, iron, manganese, and zinc in the Parkinson's disease brain. *Metallomics*. 2017;9(10):1447-1455. doi:10.1039/c7mt00244k

17. Griffiths PD, Crossman AR. Distribution of iron in the basal ganglia and neocortex in postmortem tissue in

Parkinson’s disease and Alzheimer’s disease. *Dementia*. 1993;4(2):61-65.

18. Dexter DT, Wells FR, Agid F, et al. Increased nigral iron accumulation in postmortem Parkinsonian brain. *Lancet*. 1987;2:1219-1220.

19. Zecca L, Berg D, Arzberger T, et al. In Vivo Detection of Iron and Neuromelanin by Transcranial Sonography : A New Approach for Early Detection of Substantia Nigra Damage. 2005;20(10):1278-1285. doi:10.1002/mds.20550

20. Ramos P, Santos A, Pinto NR, Mendes R, Magalhães T, Almeida A. Iron levels in the human brain: A post-mortem study of anatomical region differences and age-related changes. *J Trace Elem Med Biol*. 2014;28(1):13-17. doi:10.1016/j.jtemb.2013.08.001

21. Fernández B, Ferrer I, Gil F, Hil S. Biomonitorization of iron accumulation in the substantia nigra from Lewy body disease patients. 2017;4(February):188-193. doi:10.1016/j.toxrep.2017.03.005

22. Davies KM, Bohic S, Carmona A, et al. Copper pathology in vulnerable brain regions in Parkinson’s disease. *Neurobiol Aging*. 2014;35(4):858-866. doi:10.1016/j.neurobiolaging.2013.09.034

23. Devos D, Moreau C, Devedjian JC, et al. Targeting chelatable iron as a therapeutic modality in Parkinson’s disease. *Antioxidants Redox Signal*. 2014;21(2):195-210. doi:10.1089/ars.2013.5593

24. Grolez G, Moreau C, Sablonnière B, et al. Ceruloplasmin activity and iron chelation treatment of patients with Parkinson’s disease. *BMC Neurol*. 2015;15(1):2-7. doi:10.1186/s12883-015-0331-3

25. Martin-Bastida A, Ward RJ, Newbould R, et al. Brain iron chelation by deferiprone in a phase 2 randomised double-blinded placebo controlled clinical trial in Parkinson’s disease. *Sci Rep*. 2017;7(1):1-9. doi:10.1038/s41598-017-01402-2

26. Connor JR, Snyder BS, Arosio P, Loeffler DA, LeWitt P. A quantitative analysis of isoferritins in select regions of aged, parkinsonian, and Alzheimer’s diseased brains. *J Neurochem*. 1995;65(2):717-724. doi:10.1046/j.1471-4159.1995.65020717.x

27. Dexter DT, Carayon A, Vidailhet M, et al. Decreased Ferritin Levels in Brain in Parkinson’s Disease. *J Neurochem*. 1990;55(1):16-20. doi:10.1111/j.1471-4159.1990.tb08814.x

28. Dutta D, Ali N, Banerjee E, et al. Low Levels of Prohibitin in Substantia Nigra Makes Dopaminergic Neurons Vulnerable in Parkinson ’ s Disease. 2018:804-821. doi:10.1007/s12035-016-0328-y

29. Faucheux BA, Hauw JJ, Agid Y, Hirsch EC. The density of [125I]-transferrin binding sites on perikarya of melanized neurons of the substantia nigra is decreased in Parkinson’s disease. *Brain Res*. 1997;749(1):170-174. doi:10.1016/s0006-8993(96)01412-6

30. Faucheux BA, Hirsch EC, Villares J, et al. Distribution of 125I-Ferrotransferrin Binding Sites in the Mesencephalon of Control Subjects and Patients with Parkinson’s Disease. *J Neurochem*. 1993;60(6):2338-2341. doi:10.1111/j.1471-4159.1993.tb03527.x

31. Faucheux BA, Martin M, Beaumont C, Hauw J, Agid Y, Hirsch EC. Lack of up-regulation of ferritin is associated with sustained iron regulatory protein-1 binding activity in the substantia nigra of patients with Parkinson ’ s disease. 2002:320-330.

32. Faucheux BA, Nillesse N, Damier P, et al. Expression of lactoferrin receptors is increased in the mesencephalon of patients with Parkinson disease. *Proc Natl Acad Sci U S A*. 1995;92(21):9603-9607. doi:10.1073/pnas.92.21.9603

33. Galazka-friedman J, Bauminger ER, Koziorowski D, Friedman A. ¨ ssbauer spectroscopy and ELISA studies reveal differences between Parkinson ’ s disease and control substantia nigra. 2004;1688:130-136. doi:10.1016/j.bbadis.2003.11.005

34. Kunikowska G, Jenner P. Alterations in m-RNA expression for Cu,Zn-superoxide dismutase and glutathione peroxidase in the basal ganglia of MPTP-treated marmosets and patients with Parkinson's disease. *Brain Res*. 2003;968(2):206-218. doi:10.1016/S0006-8993(03)02240-6

35. Liu C, Zhang CW, Lo SQ, et al. S-nitrosylation of divalent metal transporter 1 enhances iron uptake to mediate loss of dopaminergic neurons and motoric deficit. *J Neurosci*. 2018;38(39):8364-8377. doi:10.1523/JNEUROSCI.3262-17.2018

36. Loeffler DA, Sima AAF, LeWitt PA. Ceruloplasmin immunoreactivity in neurodegenerative disorders. *Free Radic Res*. 2001;35(2):111-118. doi:10.1080/10715760100300651

37. Mirza B, Hadberg H, Thomsen P, Moos T. The absence of reactive astrocytosis is indicative of a unique inflammatory process in Parkinson's disease. *Neuroscience*. 1999;95(2):425-432. doi:10.1016/S0306-4522(99)00455-8

38. Poirier J, Dea D, Baccichet A, Thiffaul C. Superoxide Dismutase Expression in Parkinson's Disease. *Ann N Y Acad Sci*. 1994;738(1):116-120. doi:10.1111/j.1749-6632.1994.tb21796.x

39. Saggu H, Cooksey J, Dexter D, et al. A Selective Increase in Particulate Superoxide Dismutase Activity in Parkinsonian Substantia Nigra. *J Neurochem*. 1989;53(3):692-697. doi:10.1111/j.1471-4159.1989.tb11759.x

40. Schipper HM, Liberman A, Stopa EG. Neural Heme Oxygenase-1 Expression in Idiopathic Parkinson ' s Disease. 1998;68(150):60-68.

41. Trist BG, Davies KM, Cottam V, et al. Amyotrophic lateral sclerosis-like superoxide dismutase 1 proteinopathy is associated with neuronal loss in Parkinson's disease brain. *Acta Neuropathol*. 2017;134(1):113-127. doi:10.1007/s00401-017-1726-6

42. Trist BG, Fifita JA, Freckleton SE, et al. Accumulation of dysfunctional SOD1 protein in Parkinson's disease is not associated with mutations in the SOD1 gene. *Acta Neuropathol*. 2018;135(1):155-156. doi:10.1007/s00401-017-1779-6

43. Werner CJ, Heyny-von Haussen R, Mall G, Wolf S. Proteome analysis of human substantia nigra in Parkinson's disease. *Proteome Sci*. 2008;6:1-14. doi:10.1186/1477-5956-6-8

44. Aamodt AH, Stovner LJ, Thorstensen K, Lydersen S, White LR, Aasly JO. Prevalence of haemochromatosis gene mutations in Parkinson's disease. *J Neurol Neurosurg Psychiatry*. 2007;78(3):315-317. doi:10.1136/jnnp.2006.101352

45. Akbas N, Hochstrasser H, Deplazes J, et al. Screening for mutations of the HFE gene in Parkinson's disease patients with hyperechogenicity of the substantia nigra. *Neurosci Lett*. 2006;407(1):16-19. doi:10.1016/j.neulet.2006.07.070

46. Ayuso P, Martínez C, Pastor P, et al. An association study between heme oxygenase-1 genetic variants and Parkinson's disease. *Front Cell Neurosci*. 2014;8(SEP):1-8. doi:10.3389/fncel.2014.00298

47. Biasiotto G, Goldwurm S, Finazzi D, et al. HFE gene mutations in a population of Italian Parkinson's disease patients. *Park Relat Disord*. 2008;14(5):426-430. doi:10.1016/j.parkreldis.2007.10.011

48. Borie C, Gasparini F, Verpillat P, et al. Association study between iron-related genes polymorphisms and Parkinson's disease. *J Neurol*. 2002;249(7):801-804. doi:10.1007/s00415-002-0704-6

49. Buchanan DD, Silburn PA, Chalk JB, Le Couteur DG, Mellick GD. The Cys282Tyr polymorphism in the HFE gene in Australian Parkinson's disease patients. *Neurosci Lett*. 2002;327(2):91-94. doi:10.1016/s0304-3940(02)00398-1

50. Chen R, Langston JW, Chan P. Screening of ferritin light polypeptide 460 – 461InsA mutation in Parkinson ' s disease patients in North America. 2002;335:144-146.

51. Deplazes J, Schöbel K, Hochstrasser H, et al. Screening for mutations of the IRP2 gene in Parkinson's disease patients with hyperechogenicity of the substantia nigra. *J Neural Transm*. 2004;111(4):515-521. doi:10.1007/s00702-004-0125-z

52. Farin FM, Hitosis Y, Hallagan SE, et al. Genetic Polymorphisms of Superoxide Dismutase in Parkinson ' s Disease. 2001;16(4):705-707. doi:10.1002/mds.1153

53. Felletschin B, Bauer P, Walter U, et al. Screening for mutations of the ferritin light and heavy genes in Parkinson's disease patients with hyperechogenicity of the substantia nigra. *Neurosci Lett*. 2003;352(1):53-56. doi:10.1016/j.neulet.2003.08.026

54. Castiglioni E, Finazzi D, Goldwurm S, et al. Analysis of nucleotide variations in genes of iron management in patients of Parkinson's disease and other movement disorders. *Park Dis*. 2011;2011.

55. Foglieni B, Ferrari F, Goldwurm S, et al. Analysis of ferritin genes in Parkinson disease. *Clin Chem Lab Med*. 2007;45(11):1450-1456. doi:10.1515/CCLM.2007.307

56. Funke C, Tomiuk J, Riess O, Berg D, Soehn AS. Genetic analysis of heme oxygenase-1 (HO-1) in German Parkinson's disease patients. *J Neural Transm*. 2009;116(7):853-859. doi:10.1007/s00702-009-0237-6

57. Gasser T, Wszolek ZK, Trofatter J, et al. Genetic linkage studies in autosomal dominant parkinsonism: Evaluation of seven candidate genes. *Ann Neurol*. 1994;36(3):387-396. doi:10.1002/ana.410360310

58. Greco V, De Marco E V, Rocca FE, et al. Association study between four polymorphisms in the HFE, TF and TFR genes and Parkinson's disease in Southern Italy. *Neurol Sci*. 2011;32(3):525-527. doi:10.1007/s10072-011-0504-9

59. Guerreiro RJ, Bras JM, Santana I, et al. Association of HFE common mutations with Parkinson's disease, Alzheimer's disease and mild cognitive impairment in a Portuguese cohort. *BMC Neurol*. 2006;6. doi:10.1186/1471-2377-6-24

60. He Q, Du T, Yu X, et al. DMT1 polymorphism and risk of Parkinson's disease. *Neurosci Lett*. 2011;501(3):128-131. doi:10.1016/j.neulet.2011.07.001

61. Hochstrasser H, Tomiuk J, Walter U, et al. Functional relevance of ceruloplasmin mutations in Parkinson's disease. *FASEB J*. 2005;19(13):1851-1853. doi:10.1096/fj.04-3486fje

62. Lee PL, Gelbart T, West C, Halloran C, Sipe JC, Beutler E. Polymorphisms in Iron-Responsive Binding Protein 2 and Lack of Association with Sporadic Parkinson ' s Disease. 2002;17(6):1302-1304. doi:10.1002/mds.10253

63. Mariani S, Ventriglia M, Simonelli I, et al. Association between sex, systemic iron variation and probability of Parkinson's disease. *Int J Neurosci*. 2016;126(4):354-360. doi:10.3109/00207454.2015.1020113

64. Mariani S, Ventriglia M, Simonelli I, et al. Effects of hemochromatosis and transferrin gene mutations on peripheral iron dyshomeostasis in mild cognitive impairment and Alzheimer's and Parkinson's diseases. *Front Aging Neurosci*. 2013;5(AUG):1-7. doi:10.3389/fnagi.2013.00037

65. Parboosingh JS, Rousseau M, Rogan F, et al. Absence of Mutations in Superoxide Dismutase and Catalase Genes in Patients With Parkinson's Disease. *Arch Neurol*. 1995;52(12):1160-1163.

66. Rhodes SL, Buchanan DD, Ahmed I, et al. Neurobiology of Disease Pooled analysis of iron-related genes in Parkinson ' s disease : Association with transferrin. *Neurobiol Dis*. 2014;62:172-178. doi:10.1016/j.nbd.2013.09.019

67. Dekker MCJJ, Giesbergen PC, Njajou OT, et al. Mutations in the hemochromatosis gene (HFE), Parkinson's disease and parkinsonism. *Neurosci Lett*. 2003;348(2):117-119. doi:10.1016/s0304-3940(03)00713-4

68. Saadat SM, Değirmenci T, Özkan S, et al. Is the 1254T>C polymorphism in the DMT1 gene associated with Parkinson's disease? *Neurosci Lett*. 2015;594:51-54. doi:10.1016/j.neulet.2015.03.054

69. Tian S, Yang X, Zhao Q, et al. Association between a heme oxygenase-2 genetic variant and risk of Parkinson's disease in Han Chinese. *Neurosci Lett*. 2017;642:119-122. doi:10.1016/j.neulet.2017.02.008

70. Dexter DT, Sian J, Rose S, et al. Indices of oxidative stress and mitochondrial function in individuals with incidental Lewy body disease. *Ann Neurol*. 1994;35(1):38-44.

71. Zhao N, Xiao J, Zheng Z, et al. Single-nucleotide polymorphisms and haplotypes of non-coding area in the CP gene are correlated with Parkinson's disease. *Neurosci Bull*. 2015;31(2):245-256. doi:10.1007/s12264-014-1512-6

72. Acosta-Cabronero J, Cardenas-Blanco A, Betts MJ, et al. The whole-brain pattern of magnetic susceptibility perturbations in Parkinson's disease. *Brain*. 2017;140(1):118-131. doi:10.1093/brain/aww278

73. An H, Zeng X, Niu T, et al. Quantifying iron deposition within the substantia nigra of Parkinson's disease by quantitative susceptibility mapping. *J Neurol Sci*. 2018;386(January):46-52. doi:10.1016/j.jns.2018.01.008

74. Antonini A, Leenders KL, Meier D, Oertel WH, Boesiger P, Anliker M. T2 relaxation time in patients with Parkinson's disease. *Neurology*. 1993;43(4):697-700. doi:10.1212/wnl.43.4.697

75. Costa-Mallen P, Gatenby C, Friend S, et al. Brain iron concentrations in regions of interest and relation with serum iron levels in Parkinson disease. *J Neurol Sci*. 2017;378:38-44. doi:10.1016/j.jns.2017.04.035

76. Aquino D, Contarino V, Albanese A, et al. Substantia nigra in Parkinson ' s disease : a multimodal MRI comparison between early and advanced stages of the disease. 2014:753-758. doi:10.1007/s10072-013-1595-2

77. Arribarat G, Pasternak O, Barros A De, Galitzky M, Rascol O, Péran P. Parkinsonism and Related Disorders Substantia nigra locations of iron-content , free-water and mean di ff usivity abnormalities in moderate stage Parkinson ' s disease. *Park Relat Disord*. 2019;65(December 2018):146-152. doi:10.1016/j.parkreldis.2019.05.033

78. Atasoy HT, Nuyan O, Tunc T, Yorubulut M, Unal AE, Inan LE. T2-weighted MRI in Parkinson's disease; substantia nigra pars compacta hypointensity correlates with the clinical scores. *Neurol India*. 2004;52(3):332-337.

79. Azuma M, Hirai T, Yamada K, et al. Lateral Asymmetry and Spatial Difference of Iron Deposition in the Substantia Nigra of Patients with Parkinson Disease Measured with Quantitative Susceptibility Mapping. *AJNR Am J Neuroradiol*. 2016;37(5):782-788. doi:10.3174/ajnr.A4645

80. Barbosa JHO, Santos AC, Tumas V, et al. Quantifying brain iron deposition in patients with Parkinson's disease using quantitative susceptibility mapping, R2 and R2*. *Magn Reson Imaging*. 2015;33(5):559-565. doi:10.1016/j.mri.2015.02.021

81. Bartzokis G, Cummings JL, Markham CH, et al. MRI evaluation of brain iron in earlier- and later-onset Parkinson's disease and normal subjects. *Magn Reson Imaging*. 1999;17(2):213-222. doi:10.1016/S0730-725X(98)00155-6

82. Baudrexel S, Nurnberger L, Rub U, et al. Quantitative mapping of T1 and T2* discloses nigral and brainstem pathology in early Parkinson's disease. *Neuroimage*. 2010;51(2):512-520. doi:10.1016/j.neuroimage.2010.03.005

83. Behnke S, Schroeder U, Dillmann U, et al. Hyperechogenicity of the substantia nigra in healthy controls is related to MRI changes and to neuronal loss as determined by F-Dopa PET. *Neuroimage*. 2009;47(4):1237-1243. doi:10.1016/j.neuroimage.2009.05.072

84. Bergsland N, Zivadinov R, Schweser F, Hagemeier J, Lichter D, Guttuso T. Ventral posterior substantia nigra iron increases over 3 years in Parkinson's disease. *Mov Disord*. 2019;34(7):1006-1013. doi:10.1002/mds.27730

85. Bunzeck N, Singh-curry V, Eckart C, et al. Parkinsonism and Related Disorders Motor phenotype and magnetic resonance measures of basal ganglia iron levels in Parkinson ' s disease q. 2013;19:1136-1142. doi:10.1016/j.parkreldis.2013.08.011

86. Chen Q, Chen Y, Zhang Y, et al. Iron deposition in Parkinson's disease by quantitative susceptibility mapping. *BMC Neurosci*. 2019;20(1):1-8. doi:10.1186/s12868-019-0505-9

87. Dashtipour K, Liu M, Kani C, et al. Iron accumulation is not homogenous among patients with Parkinson's disease. *Parkinsons Dis*. 2015;2015. doi:10.1155/2015/324843

88. Du G, Lewis MM, Shaffer ML, et al. Serum Cholesterol and Nigrostriatal R2 * Values in Parkinson ' s Disease. 2012;7(4):1-7. doi:10.1371/journal.pone.0035397

89. Du G, Lewis MM, Styner M, Shaffer ML. Combined R2 * and Diffusion Tensor Imaging Changes in the Substantia Nigra in Parkinson ' s Disease. 2011;26(9):1627-1632. doi:10.1002/mds.23643

90. Du G, Liu T, Lewis MM, et al. Quantitative susceptibility mapping of the midbrain in Parkinson's disease. *Mov Disord*. 2016;31(3):317-324. doi:10.1002/mds.26417

91. Esterhammer R, Seppi K, Reiter E, et al. Potential of diffusion tensor imaging and relaxometry for the detection of specific pathological alterations in Parkinson's Disease (PD). *PLoS One*. 2015;10(12):1-18. doi:10.1371/journal.pone.0145493

92. Ghassaban K, He N, Sethi SK, Huang P, Chen S. Regional High Iron in the Substantia Nigra Differentiates Parkinson ' s Disease Patients From Healthy Controls. 2019;11(May):1-10. doi:10.3389/fnagi.2019.00106

93. Gorell JM, Ordidge RJ, Brown GG, Deniau JC, Buderer NM, Helpern JA. Increased iron-related MRI contrast in the substantia nigra in Parkinson's disease. *Neurology*. 1995;45(6):1138-1143. doi:10.1212/wnl.45.6.1138

94. Graham JM, Paley MN, Grünewald RA, Hoggard N, Griffiths PD. Brain iron deposition in Parkinson's disease imaged using the PRIME magnetic resonance sequence. *Brain*. 2000;123 Pt 12:2423-2431. doi:10.1093/brain/123.12.2423

95. Guan X, Xuan M, Gu Q, et al. Regionally progressive accumulation of iron in Parkinson's disease as measured by quantitative susceptibility mapping. *NMR Biomed*. 2015;30(4):e3489. doi:10.1002/nbm.3489

96. Guan X, Xuan M, Gu Q, et al. Influence of regional iron on the motor impairments of Parkinson's disease: A quantitative susceptibility mapping study. *J Magn Reson Imaging*. 2017;45(5):1335-1342. doi:10.1002/jmri.25434

97. Guan X, Zhang Y, Wei H, et al. Iron-related nigral degeneration influences functional topology mediated by striatal dysfunction in Parkinson's disease. *Neurobiol Aging*. 2019;75:83-97. doi:10.1016/j.neurobiolaging.2018.11.013

98. He N, Ling H, Ding B, et al. Region-specific disturbed iron distribution in early idiopathic Parkinson's disease measured by quantitative susceptibility mapping. *Hum Brain Mapp*. 2015;36(11):4407-4420. doi:10.1002/hbm.22928

99. Homayoon N, Pirpamer L, Franthal S, et al. Nigral iron deposition in common tremor disorders. *Mov Disord*. 2019;34(1):129-132. doi:10.1002/mds.27549

100. Hopes L, Grolez G, Moreau C, et al. Magnetic Resonance Imaging Features of the Nigrostriatal System: Biomarkers of Parkinson's Disease Stages? *PLoS One*. 2016;11(4):e0147947.

doi:10.1371/journal.pone.0147947

101. Huang Y-W, Jeng J-S, Tsai C-F, Chen L-L, Wu R-M. Transcranial imaging of substantia nigra hyperechogenicity in a Taiwanese cohort of Parkinson's disease. *Mov Disord*. 2007;22(4):550-555. doi:10.1002/mds.21372

102. Ji S, Zhang S, Mao Z, et al. Quantitative assessment of iron deposition in Parkinson's disease using enhanced T2 star-weighted angiography. *Neurol India*. 2016;64(3):428-435. doi:10.4103/0028-3886.181532

103. Jin L, Wang J, Jin H, et al. Nigral iron deposition occurs across motor phenotypes of Parkinson's disease. *Eur J Neurol*. 2012;19(7):969-976. doi:10.1111/j.1468-1331.2011.03658.x

104. Jin L, Wang J, Zhao L, et al. Decreased serum ceruloplasmin levels characteristically aggravate nigral iron deposition in Parkinson ' s disease. 2011;(2010):50-58. doi:10.1093/brain/awq319

105. Kim T-H, Lee J-H. Serum uric acid and nigral iron deposition in Parkinson's disease: a pilot study. *PLoS One*. 2014;9(11):e112512. doi:10.1371/journal.pone.0112512

106. Kosta P, Argyropoulou MI, Markoula S, Konitsiotis S. MRI evaluation of the basal ganglia size and iron content in patients with Parkinson's disease. *J Neurol*. 2006;253(1):26-32. doi:10.1007/s00415-005-0914-9

107. Langley J, Huddleston DE, Sedlacik J, Boelmans K, Hu XP. Parkinson's disease-related increase of T2*-weighted hypointensity in substantia nigra pars compacta. *Mov Disord*. 2017;32(3):441-449. doi:10.1002/mds.26883

108. Lewis MM, Du G, Kidacki M, et al. Higher iron in the red nucleus marks Parkinson's dyskinesia. *Neurobiol Aging*. 2013;34(5):1497-1503. doi:10.1016/j.neurobiolaging.2012.10.025

109. Liu Z, Shen HC, Lian TH, et al. Iron deposition in substantia nigra: Abnormal iron metabolism, neuroinflammatory mechanism and clinical relevance. *Sci Rep*. 2017;7(1):1-7. doi:10.1038/s41598-017-14721-1

110. Lotfipour AK, Wharton S, Schwarz ST, et al. High resolution magnetic susceptibility mapping of the substantia nigra in Parkinson's disease. *J Magn Reson Imaging*. 2012;35(1):48-55. doi:10.1002/jmri.22752

111. Martin WRW, Wieler M, Gee M. Midbrain iron content in early Parkinson disease: A potential biomarker of disease status. *Neurology*. 2008;70(16 PART 2):1411-1417. doi:10.1212/01.wnl.0000286384.31050.b5

112. Martin-Bastida A, Lao-Kaim NP, Loane C, et al. Motor associations of iron accumulation in deep grey matter nuclei in Parkinson's disease: a cross-sectional study of iron-related magnetic resonance imaging susceptibility. *Eur J Neurol*. 2017;24(2):357-365. doi:10.1111/ene.13208

113. Martínez-Hernández R, Montes S, Higuera-Calleja J, et al. Plasma ceruloplasmin ferroxidase activity correlates with the nigral sonographic area in Parkinson's disease patients: A pilot study. *Neurochem Res*. 2011;36(11):2111-2115. doi:10.1007/s11064-011-0535-x

114. Han YH, Lee JH, Kang BM, et al. Topographical differences of brain iron deposition between progressive supranuclear palsy and parkinsonian variant multiple system atrophy. *J Neurol Sci*. 2013;325(1-2):29-35. doi:10.1016/j.jns.2012.11.009

115. Michaeli S, Oz G, Sorce DJ, et al. Assessment of brain iron and neuronal integrity in patients with Parkinson's disease using novel MRI contrasts. *Mov Disord*. 2007;22(3):334-340. doi:10.1002/mds.21227

116. Murakami Y, Kakeda S, Watanabe K, et al. Usefulness of quantitative susceptibility mapping for the diagnosis of Parkinson disease. *AJNR Am J Neuroradiol*. 2015;36(6):1102-1108. doi:10.3174/ajnr.A4260

117. Naduthota RM, Honnedevasthana AA, Lenka A, et al. Association of freezing of gait with nigral iron

accumulation in patients with Parkinson's disease. *J Neurol Sci*. 2017;382(September):61-65. doi:10.1016/j.jns.2017.09.033

118. Nestrasil I, Michaeli S, Liimatainen T, et al. T1ρ and T2ρ MRI in the evaluation of parkinson's disease. *J Neurol*. 2010;257(6):964-968. doi:10.1007/s00415-009-5446-2

119. Peckham ME, Dashtipour K, Holshouser BA, et al. Novel Pattern of Iron Deposition in the Fascicula Nigrale in Patients with Parkinson's Disease: A Pilot Study. *Radiol Res Pract*. 2016;2016:1-7. doi:10.1155/2016/9305018

120. Péran P, Cherubini A, Assogna F, et al. Magnetic resonance imaging markers of Parkinson's disease nigrostriatal signature. *Brain*. 2010;133(11):3423-3433. doi:10.1093/brain/awq212

121. Pyatigorskaya N, Sharman M, Corvol JC, et al. High nigral iron deposition in LRRK2 and Parkin mutation carriers using R2* relaxometry. *Mov Disord*. 2015;30(8):1077-1084. doi:10.1002/mds.26218

122. Qiao PF, Shi F, Jiang MF, Gao Y, Niu GM. Application of high-field magnetic resonance imaging in Parkinson's disease. *Exp Ther Med*. 2017;13(5):1665-1670. doi:10.3892/etm.2016.3551

123. Reimão S, Ferreira S, Nunes RG, et al. Magnetic resonance correlation of iron content with neuromelanin in the substantia nigra of early-stage Parkinson's disease. *Eur J Neurol*. 2016;23(2):368-374. doi:10.1111/ene.12838

124. Rossi M, Ruottinen H, Soimakallio S, Elovaara I, Dastidar P. Clinical MRI for iron detection in Parkinson's disease. *Clin Imaging*. 2013;37(4):631-636. doi:10.1016/j.clinimag.2013.02.001

125. Rossi ME, Ruottinen H, Saunamäki T, Elovaara I, Dastidar P. Imaging Brain Iron and Diffusion Patterns. A Follow-up Study of Parkinson's Disease in the Initial Stages. *Acad Radiol*. 2014;21(1):64-71. doi:10.1016/j.acra.2013.09.018

126. Ryvlin P, Broussolle E, Piollet H, Viallet F, Khalfallah Y, Chazot G. Magnetic resonance imaging evidence of decreased putamenal iron content in idiopathic Parkinson's disease. *Arch Neurol*. 1995;52(6):583-588. doi:10.1001/archneur.1995.00540300057013

127. Schwarz ST, Mougin O, Xing Y, et al. Parkinson's disease related signal change in the nigrosomes 1–5 and the substantia nigra using T2* weighted 7T MRI. *NeuroImage Clin*. 2018;19(April):683-689. doi:10.1016/j.nicl.2018.05.027

128. Sethi SK, Kisch SJ, Ghassaban K, et al. Iron quantification in Parkinson's disease using an age-based threshold on susceptibility maps: The advantage of local versus entire structure iron content measurements. *Magn Reson Imaging*. 2019;55:145-152. doi:10.1016/j.mri.2018.10.001

129. Shahmaei V, Faeghi F, Mohammdbeigi A, Hashemi H, Ashrafi F. Evaluation of iron deposition in brain basal ganglia of patients with Parkinson's disease using quantitative susceptibility mapping. *Eur J Radiol Open*. 2019;6(January):169-174. doi:10.1016/j.ejro.2019.04.005

130. Shin C, Lee S, Lee JY, Rhim JH, Park SW. Non-Motor Symptom Burdens Are Not Associated with Iron Accumulation in Early Parkinson's Disease: a Quantitative Susceptibility Mapping Study. *J Korean Med Sci*. 2018;33(13):e96. doi:10.3346/jkms.2018.33.e96

131. Sjöström H, Granberg T, Westman E, Svenningsson P. Quantitative susceptibility mapping differentiates between parkinsonian disorders. *Park Relat Disord*. 2017;44:51-57. doi:10.1016/j.parkreldis.2017.08.029

132. Tambasco N, Paolini F, Chiappiniello A, et al. Neurobiology of Aging T2 * -weighted MRI values correlate with motor and cognitive dysfunction in Parkinson ' s disease. *Neurobiol Aging*. 2019;80:91-98. doi:10.1016/j.neurobiolaging.2019.04.005

133. Uchida Y, Kan H, Sakurai K, et al. Voxel-based quantitative susceptibility mapping in Parkinson's disease

with mild cognitive impairment. *Mov Disord*. 2019;34(8):1164-1173. doi:10.1002/mds.27717

134. Ulla M, Bonny JM, Ouchchane L, Rieu I, Claise B, Durif F. Is R2* a new MRI biomarker for the progression of Parkinson's disease? A longitudinal follow-up. *PLoS One*. 2013;8(3):e57904. doi:10.1371/journal.pone.0057904

135. Vymazal J, Righini A, Brooks RA, et al. T1 and T2 in the brain of healthy subjects, patients with Parkinson disease, and patients with multiple system atrophy: relation to iron content. *Radiology*. 1999;211(2):489-495. doi:10.1148/radiology.211.2.r99ma53489

136. Wallis LI, Paley MNJ, Graham JM, et al. MRI assessment of basal ganglia iron deposition in Parkinson's disease. *J Magn Reson Imaging*. 2008;28(5):1061-1067. doi:10.1002/jmri.21563

137. Wang C, Fan G, Xu K, Wang S. Quantitative assessment of iron deposition in the midbrain using 3D-enhanced T2 star weighted angiography (ESWAN): a preliminary cross-sectional study of 20 Parkinson's disease patients. *Magn Reson Imaging*. 2013;31(7):1068-1073. doi:10.1016/j.mri.2013.04.015

138. Wieler M, Gee M, Camicioli R, Martin WRW. Freezing of gait in early Parkinson's disease: Nigral iron content estimated from magnetic resonance imaging. *J Neurol Sci*. 2016;361:87-91. doi:10.1016/j.jns.2015.12.008

139. Wieler M, Gee M, Martin WRW. Longitudinal midbrain changes in early Parkinson's disease: Iron content estimated from R2*/MRI. *Park Relat Disord*. 2015;21(3):179-183. doi:10.1016/j.parkreldis.2014.11.017

140. Wu SF, Zhu ZF, Kong Y, et al. Assessment of cerebral iron content in patients with Parkinson's disease by the susceptibility-weighted MRI. *Eur Rev Med Pharmacol Sci*. 2014;18(18):2605-2608.

141. Xuan M, Guan X, Gu Q, et al. Different iron deposition patterns in early- and middle-late-onset Parkinson's disease. *Park Relat Disord*. 2017;44:23-27. doi:10.1016/j.parkreldis.2017.08.013

142. Zhang J, Zhang Y, Wang J, et al. Characterizing iron deposition in Parkinson's disease using susceptibility-weighted imaging: An in vivo MR study. *Brain Res*. 2010;1330:124-130. doi:10.1016/j.brainres.2010.03.036

143. Zhang W, Sun SG, Jiang YH, Qiao X, Sun X, Wu Y. Determination of brain iron content in patients with Parkinson's disease using magnetic susceptibility imaging. *Neurosci Bull*. 2009;25(6):353-360. doi:10.1007/s12264-009-0225-8

144. Langkammer C, Pirpamer L, Seiler S, et al. Quantitative susceptibility mapping in Parkinson's disease. *PLoS One*. 2016;11(9):1-13. doi:10.1371/journal.pone.0162460

145. Li G, Zhai G, Zhao X, et al. NeuroImage 3D texture analyses within the substantia nigra of Parkinson ' s disease patients on quantitative susceptibility maps and R2 * maps. *Neuroimage*. 2019;188(October 2018):465-472. doi:10.1016/j.neuroimage.2018.12.041

146. Sakurai K, Imabayashi E, Tokumaru AM, et al. Volume of interest analysis of spatially normalized PRESTO imaging to differentiate between parkinson disease and atypical Parkinsonian syndrome. *Magn Reson Med Sci*. 2017;16(1):16-22. doi:10.2463/mrms.mp.2015-0132

147. Schmidauer C, Sojer M, Seppi K, et al. Transcranial ultrasound shows nigral hypoechogenicity in restless legs syndrome. *Ann Neurol*. 2005;58(4):630-634. doi:10.1002/ana.20572

148. Takahashi H, Watanabe Y, Tanaka H, et al. Comprehensive MRI quanti fi cation of the substantia nigra pars compacta in Parkinson ' s disease. *Eur J Radiol*. 2018;109(May):48-56. doi:10.1016/j.ejrad.2018.06.024

149. Ward PGD, Harding IH, Close TG, et al. Longitudinal evaluation of iron concentration and atrophy in the dentate nuclei in friedreich ataxia. *Mov Disord*. 2019;34(3):335-343. doi:10.1002/mds.27606

150. Aschermann Z, Perlaki G, Orsi G, et al. Quantitative assessment of brain iron by R2* relaxometry in patients

with cervical dystonia. *Mov Disord*. 2015;30(10):1422-1426. doi:10.1002/mds.26306

151. Ariza J, Rogers H, Hartvigsen A, et al. Iron accumulation and dysregulation in the putamen in fragile X-associated tremor/ataxia syndrome. *Mov Disord*. 2017;32(4):585-591. doi:10.1002/mds.26902

152. Deguchi K, Ikeda K, Kume K, et al. Significance of the hot-cross bun sign on T2*-weighted MRI for the diagnosis of multiple system atrophy. *J Neurol*. 2015;262(6):1433-1439. doi:10.1007/s00415-015-7728-1

153. Lee J-H, Han Y-H, Kang B-M, Mun C-W, Lee S-J, Baik S-K. Quantitative assessment of subcortical atrophy and iron content in progressive supranuclear palsy and parkinsonian variant of multiple system atrophy. *J Neurol*. 2013;260:2094-2101. doi:10.1007/s00415-013-6951-x

154. Lee J, Kim TKCMT, Han Y. Progression of subcortical atrophy and iron deposition in multiple system atrophy : a comparison between clinical subtypes. *J Neurol*. 2015;262(8):1876-1882. doi:10.1007/s00415-015-7785-5

155. Cantuti-Castelvetri I, Keller-McGandy CE, Albers DS, et al. Expression and activity of antioxidants in the brain in progressive supranuclear palsy. *Brain Res*. 2002;930(1-2):170-181. doi:10.1016/S0006-8993(02)02244-8
